# A constrained optimization model for the provision of services in a 5G network with multi-level cybersecurity investments

**DOI:** 10.1007/s00500-022-07117-5

**Published:** 2022-05-03

**Authors:** Giorgia M. Cappello, Gabriella Colajanni, Patrizia Daniele, Daniele Sciacca

**Affiliations:** Department of Mathematics and Computer Science, University of Catania, Viale A. Doria 6, Catania, Italy

**Keywords:** 5G Network slicing, Constrained optimization, Cybersecurity, Modified genetic algorithm

## Abstract

In this paper, we present a multi-tiered network-based optimization model describing the provision of services by network slices of 5G-Service providers (e.g. through Unmanned Aerial Vehicles (UAVs) organized as Flying Ad hoc Networks (FANET)), taking into account the security levels of each provider. The three levels of the network consist of the infrastructure layers, which contain resources needed to execute a service, the slices layer, where services are served for the services layer, which represents the upper layer of the network and consists of services or applications required by users or devices. The objective of the proposed model is to establish the optimal flows between network layers and the optimal security levels in order to maximize the providers’ profits, given by the difference between the revenues obtained by the sale of services and the rental of their resources and the costs. Numerical experiments are performed and solved with a new nature-inspired genetic algorithm adapted to the optimization 5G network problem.

## Introduction

COVID-19 pandemic rapidly spread around the world affecting almost all countries and 179 million people, including 3 million deaths (xxx 2021) and raising enormous health, economic and social challenges. The strong containment measures, such as the nationwide lockdowns and the social distancing norms, led to a surge in the Internet traffic demands (Feldmann et al. 2021) and in the use of digital technologies in the daily lives. Many firms, companies and educational institutions (Zheng et al. 2020) shift to work-from-home (WFH), telehealth and telemedicine services allowing patients to receive advice and care at a distance, making it safer for all concerned (Kaplan 2020), and so on. This inevitable surge to adapt and overcome the current exceptional situation is an evidence of the digital acceleration process (Pandey and Pal 2020). The 5G communications, and in general last technological advancements, can play a vital role to tackle and address the wide spectrum of challenges due to COVID-19 (Alharbi and Rahman 2021) supporting, for example, large-scale heterogeneous traffic and users (Siriwardhana et al. 2020). In particular, in this context, the recent developments in UAV technologies provide us with multiple benefits during the emergency in public health, such as the COVID-19 pandemic ensuring reduced human contact and can also being used to enter otherwise inaccessible places (Ly and Ly 2021). Furthermore, Internet of Things (IoT), the digital transformation of organizations, cities (Manimuthu et al. 2021), society overall and the need to support a variety of vertical industries (xxx (2016)) such as manufacturing, automotive, healthcare, energy, media and entertainment are the main driver reasons of 5G systems (xxx (2018)). More specifically, such vertical industries often generate different traffic types that impose very diverse and extreme requirements than existing services do nowadays (Banchs et al. 2019). Indeed, the upgraded generation of wireless technologies 5G revolutionizes the network service architecture with the aim of meeting various user quality of service (QoS) requirements in different application scenarios (Osseiran et al. 2014). The new performance criteria required for the new applications and business models in the future IoT include massive connectivity, security, trustworthy, coverage of wireless communication, ultra-low latency, ultra-reliable, through-put, and so on, for huge number of IoT devices (Li et al. 2018). Network slicing is one key technology that differentiates 5G from 4G. By slicing a physical network into several logical networks, network slicing can support on-demand tailored services for distinct application scenarios while using the same physical network. Supported by network slicing, network resources can be dynamically and efficiently allocated to logical network slices according to the corresponding QoS demands (Zhang et al. 2017).

In a fully connected 5G society, the limitations of time and space to create all-dimensional user-centered or service centric interconnections between people and things are greatly minimized (Zhang et al. 2016), and this underscores the need for robust security mechanisms across all network segments of the 5G (Ahmad et al. 2019). For example, IoT implementation impacts on reducing healthcare costs and improves treatment outcome of the COVID-19 patients (Singh et al. 2020), but the increased connectivity to existing computer networks exposed medical devices to new cybersecurity vulnerabilities. Healthcare is an attractive target for cybersecurity breaches, that include stealing health information and ransomware attacks on hospitals, and could include attacks on implanted medical devices (Abounassar et al. 2022; Coventry and Branley 2018). The introduction of new technologies, such as UAVs, and architecture, such as network slicing, makes therefore the security and privacy protection for 5G more challenging (Zhang et al. 2019). In particular, because of resource sharing among slices, security in network slicing is a critical issue that needs be addressed. Network slices serving different types of services may have different levels of security policy requirements. Therefore, it is necessary to consider that the cyberattack to one slice level in the 5G network impacts on other slices and on entire network systems (Li et al. 2017).

In this paper, we provide a Network Slicing 5G architecture suitable for creating a multi-service network (i.e. capable of providing several services) and a multi-provider network (Colajanni et al. 2022; Colajanni and Sciacca 2021). We include in our analysis the cybersecurity vulnerabilities of the 5G networks. Particularly, we consider the damage to be paid in the event that a cyberattack is successful and that depends on the security level of the provider. We develop a system-optimization problem with the aim to determine the optimal flows between the network layers that maximize the objective function consisting of the profit of all providers. We also determine the optimal security levels of the network’s providers which minimize the expected financial damage in case of successful cyberattacks.

The paper is organised as follows. Section 2 reviews the related work and explains our contributions. In the third Section, we describe the 5G network slicing architecture. In Sect. 4, we present the mathematical model and derive the nonlinear constrained optimization problem. In Sect. 5 , we outline a heuristic approach to solve realistic instances of the optimization problem proposed in this paper. The presented algorithm is tested and compared against an exact method and the standard genetic algorithm in order to configure the optimal parameters and to assess the heuristic algorithm. In Sect. 6, we summarize our results, present our conclusions, and provide suggestions for future research.

## Literature review and contributions

In the literature, the security in 5G and the related challenges as well as the use of a heuristic approach in 5G-network-based models are of particular concern and very recent topics. We divide the related work in the following two categories: optimization models on 5G services or UAV network and security in 5G networks. Moreover, in this section we explain in a detailed and punctual manner our contributions in these fields of application.

### Optimization models on 5G services or UAV networks

In the existing literature, various optimization models regarding the provision of 5G services have been proposed. Addad et al. in Addad et al. (2018) propose a MILP optimization model that enables a cost-optimal deployment of network slices, allowing a Mobile Network Operator to efficiently allocate the underlying layer resources according to the users’ requirements. For each network slice, the proposed solution guarantees the required delay and the bandwidth, while efficiently handling the usage of underlying nodes, which leads to reduced cost. The objective function of the proposed model aims to minimize the number of nodes hosting the Network Functions that constitute different network slices under placement, resources, links arrangements, latency aware and bandwidth aware constraints. In Di Puglia Pugliese et al. (2021), Di Puglia et al. address the problem of delivering parcels in a urban area, within a given time horizon, by conventional vehicles, i.e. trucks, equipped with drones. Focusing on the energy consumption of the drones, they address the problem under the field of robust optimization, thus preventing energy disruption in the worst case, minimizing the total transportation cost. Fan et al. in Fan et al. (2021) study a UAVs system task assignment model (see Macrina et al. (2020) for an extensive review on the use of drones in various applications, especially in routing problems in the context of parcel delivery) with multiple constraints and propose a discrete adaptive search whale optimization algorithm to solve it. In Fendt et al. (2018), the authors provide a standardized and easy to understand Integer Linear Program for offline mobile network slice embedding, especially focusing on resource allocation and virtual node as well as link mapping. The objective of the proposed model is to maximize the weighted sum of all embedded network slices. Finally, a simple configuration is solved using SCPSolver, a Java interface for integer linear programming (ILP) which is based on the GLPK (GNU Linear Programming Kit). In Skondras et al. (2021), Skondras et al. propose a network slicing scheme for 5G vehicular networks that aims to optimize the performance of modern network services. In particular, the proposed network architecture consists of UAVs acting as aerial relay nodes (ARNs) and road side units (RSUs) that provide communication resources to vehicular users. Moreover, the position of each ARN is optimized by applying the proposed icosagonal fuzzy TOPSIS (IFT) algorithm. In addiction, the satisfaction grade of each user service is monitored considering both the QoS and the signal-to-noise plus interference (SINR) factors. In Zhang et al. (2018), an integer optimization for the Network Function Virtualization (NFV) placement and chaining problem is formulated and it is mapped to min-cost flow problem. In this paper, authors relax the integer optimization into a linear program and propose efficient algorithms by selecting a small number of min-cost flow problems. In Gao et al. (2021), a new multi-UAV reconnaissance task allocation model is proposed. The objective function consists on minimizing the weighted sum of the total UAV consumption and the task execution time. A new heuristic algorithm, called grouping ant colony optimization algorithm, is proposed for this new model and compared with the traditional one. Authors in Giagkos et al. (2021) analyse the coordination of network-enabled UAVs that provide communication coverage to multiple mobile users on the ground (with the object of maximizing the set of mobiles covered by UAVs by balancing the power consumption); they propose also a genetic algorithm and a non-cooperative game approach to generate flying trajectories. Authors in Murray and Raj (2020) formulate a multiple flying sidekicks traveling salesman problem as an MILP problem, where customer parcels may be delivered by different UAVs and a single delivery truck. The authors determine the route of the delivery truck in order to minimize the time required to deliver all parcels and return to the depot (i.e. to minimize the makespan). A three-phased iterative heuristic is proposed that consists of solving a sequence of three subproblems. Analysis of numerical examples shows that adding more UAVs to an existing fleet tends to have diminishing marginal makespan improvements.

Ramirez et al. in Ramirez-Atencia et al. (2017) present a new multi-objective genetic algorithm for solving complex mission planning problems involving a team of UAVs and a set of ground control stations. According to this new approach, the constraints of the problem have been applied as penalty functions in the evaluation phase of the genetic algorithm.

Therefore, a lot of authors in their papers studied several optimization models inherent to 5G networks and/or networks consisting of UAVs (such as drones). However, none of the above works deal with a generic multi-level architecture that includes also external resources and exclusive customers. Moreover, in this paper we study an optimization model with the aim of maximizing the providers’ profits that allows us to determine if it is suitable to rent out/use part of own/other resources, which slices to create, what service to provide, and in what quantity, and the security levels. Furthermore, we propose here a new heuristic approach different from those present in the literature which appears to be more appropriate for the proposed model.

### Security in 5G networks

As mentioned in the Introduction, the new 5G technologies scenarios have a variety of specific requirements, bringing new vulnerabilities and thus imposing new security requirements. In Zhang et al. (2019), the authors, making an extensive review of the state of the art, identify typical security and privacy issues to be solved in 5G. They also discuss potential solutions to secure 5G networks from several perspectives, including the overall 5G security framework, core network, radio access network, cloud infrastructure, and the Internet of things (see also Ahmad et al. 2018). In Park et al. (2021), the authors provide the existing solutions in 5G networks for the different attacks detailed in various categories such as target component, technological impact, and privacy; they also present various applications and services of 5G considering the security requirements and solutions. Cybersecurity on UAVs is a timely and urgent topic and the increasing use of UAVs for inspecting critical infrastructures motivates the research interest on it (Krishna and Murphy 2017). In Krishna and Murphy (2017), the authors survey the scientific and trade literature on cybersecurity for UAV, concentrating on actual and simulated attacks, and the implications for small UAVs. In Tran (2021), the author investigates the unmanned aircraft system (UAS) cybersecurity in different aspects and presents a methodology to reinforce the cybersecurity of an existing or pre-defined UAS. In Gaurav et al. (2022), the authors propose a fog-based DDoS detection approach that uses fuzzy logic to differentiate attack traffic from normal traffic in 5G-enabled smart cities. They describe the DDoS attack at VANET (Vehicular Ad Hoc Network (VANET)) systems that is one of the cyber-attacks that attack the availability of such systems, since the vehicle nodes are not capable of exchanging valuable information. In Veerabathiran et al. (2020), the authors focus on security in a cloud computing environment, providing a homomorphic proxy re-encryption that enables various cloud users to share INFO that they redistributed HPRE encrypted utilizing their PubKs with the plausibility by a close procedure such as INFO remotely. Thereby, precision of assessment results in cloud computing environment security risk assessment to take care of the issue of the multifaceted nature of the system and the classified fuzzy cloud method applied to cloud computing environment chance ID stage that captures the cloud computing environment risk factors through a complete investigation of cloud computing environment security area.

Although previous works have underlined the security implications of 5G networks qualitatively, in this paper, we provide a quantitative mechanism, in the form of probabilities, that, when applied, guarantees the maximization of the profit of all providers in the network and the optimal security levels of the network’s providers. This is very important, since it enables providers to minimize the expected financial damage in case of successful cyberattacks.

### Our contributions

The main contributions of our paper can be summarized as follows:

- We provide a mathematical optimization model that allows us to maximize the providers’ profits in which we take into account, not only the revenues of each provider for each service (which here depend both on the vector of flows of service provided by all slices of all providers and on provider’s security level) but also the transport/transmission costs, rental costs and gains, utilization/execution costs incurred by each provider to use/execute all his resources and slices, investment costs to increase the security levels, the damage to be paid (or the refund to be received) due to an attack to a used resource or slice or to a link;
- We consider a security framework of the proposed 5G network, supposing that the security level of each provider, and, therefore, its cybersecurity vulnerability, depends on the security levels of its own slices and resources, on the security levels of its links and on its own performed activities (in resource nodes, in resource-slice links, in slice nodes and in slice-service links). Moreover, we consider cybersecurity investment costs and, furthermore, we take into account the expected losses associated with a cyberattack. In addition, we take into account a nonlinear budget constraint on investment costs in cybersecurity;
- We propose a new heuristic approach in which we have appropriately modified all the fundamental phases of the genetic algorithm, namely, generation, selection, crossover and mutation.

## 5G network description

The 5G network slicing, as mentioned above, is a network architecture that allows us to define on the same physical infrastructure a set of independent logical and/or virtual networks capable of operating simultaneously, at full efficiency and without interference, as if each of them had a dedicated physical network. Hence, each “slice” of the network is a complete network specially tailored to meet all the requirements of a particular service or application. Such a network enables, with a high level of automation, to implement and manage as independent scalable and flexible network slices that rely on the same common physical infrastructure. Each network partitioning is managed by a specific services Provider who rents and/or rents out physical resources, often sharing the same physical network with other providers. Note that an external infrastructure provider who leases its physical resources to the services providers could also exists (as the IaaS in cloud computing, see Colajanni and Daniele 2019). Depending on the availability of rented and of his own resources, each services provider can create its own customized “network slices” or adapt them to the various services or applications (hereinafter simply referred to as services) offered to the users or devices.

**Fig. 1:**
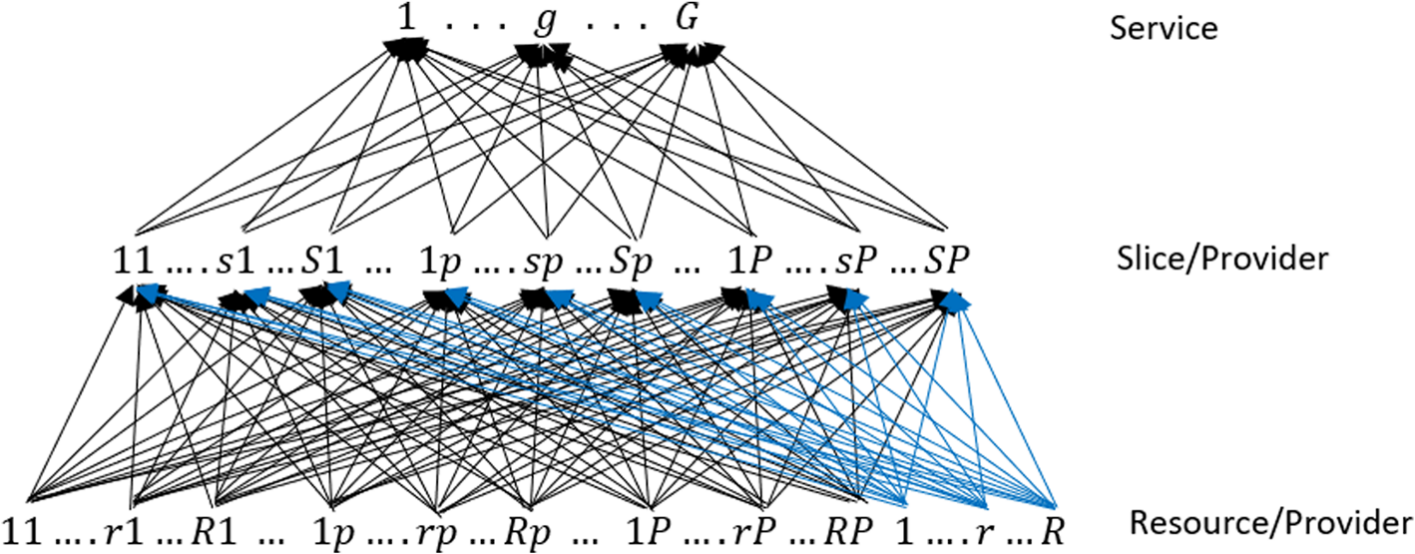
Network Topology

Although in the literature and in applied fields there are several structures of network slice architectures studied or used, it is possible to define all the elements which are common to each solution in a general and unified architecture. The 5G network slicing overall architecture can be considered as a multi-level architecture which consists of three layers, where each one contributes with its own management functions, as described below:

- Infrastructure layer: The lowest layer of the network slice architectures is composed by network resources (but also network functions) such as storage, processing, transmission nodes. This layer provides the physical 5G network resources to host the several network functions composing each slice.
- Network Slices layer: The middle layer consists of slices, where a slice can serve one or more services requested from the upper layer. The same resource (of the lowest layer) can be simultaneously shared by different network slices.
- Services layer: The upper layer consists of services or applications required by users or devices and offered by Services Providers. Each service needs to be run on a specific slice and requires specific portions of certain resources.

The Network Slicing architecture described above is not only suitable for creating a multi-service network (i.e. capable of providing several 5G services), but also a multi-provider network. Indeed, different providers will be able to share (or not) the same physical network infrastructure, on which their own virtual network slices can operate and provide the various services to customers. Furthermore, as previously mentioned, in this paper we assume we have the opportunity to extend the 5G network through the use of some UAVs, which allow the providers to reach remote zones or rural geographical areas, even not covered by electricity grid (see Faraci et al. 2019 and Grasso et al. 2021). Indeed, each node of the supply chain network could be represented by an UAV and, therefore, it is possible to extend the 5G network equipping each UAV with a computing element and thanks to the virtualization of the physical resources, the network function virtualization (NFV) and multi-access edge computing (MEC) paradigms (see Grasso and Schembra 2019). Note that the services providers are the network controllers (or orchestrators) who interface with each layer to efficiently manage the coordination between the aforementioned layers. The supply chain network, consisting of resources, slices and services, is depicted in Fig. 1. The typical 5G-services provider is denoted by $p,\; p=1,\ldots , P$ and could offer *G* types of 5G services (network services or applications). Each 5G service $g,\; g=1,\ldots , G$ provided by the services provider *p* is executed on a specific slice appropriately created by *p*. We denote by $s,\; s=1,\ldots , S$, the general slice. Therefore, the second tier of the network represents the *slice/service provider* combinations. As mentioned above, each slice (of each provider) needs one or more resources of the lowest layer and different slices can share the same resources types. We denote by $r,\; r=1,\ldots , R$, the general resource. Therefore, the lower tier of the network represents the *resource/service provider* combinations to which we add the external resources made available by the IaaS providers. We will handle the resources of external IaaS providers as them of the $P+1$ provider. Observe that all the resources of all the services providers and Infrastructure service providers can be rent and used by each services provider.

Note that, as mentioned above, a fleet of UAVs, interconnected each other via 5G technology and organized as a FANET, could constitute the node set of the supply chain network. Moreover, in this paper, driven by realty, we assume that there are some exclusive customers (intended as users or devices requiring the services) of some providers. Hence, part of the demands for services must be satisfied by the providers who have entered into an agreement with such exclusive customers. Obviously, no more service can be provided than requested, resources are limited and slices have a maximum execution capacity that cannot be exceeded.

Furthermore, in this paper we take into account another main aspect of the 5G networks: the security. Particularly, we consider the damage to be paid in the event that a cyberattack is successful and that depends on the security level of the provider. Furthermore, we consider some investment costs to increase the security levels and assume that these costs are less than the maximum budget that the provider decides to invest. The objective is to establish the optimal flows (between the network layers) and the optimal security level in order to maximize the providers’ profits (given by the difference between the revenues and the costs). In such a way, for each services provider, we obtain:

- If it is suitable to rent out part of his resources (if so, the amount of each resource to be leased);
- If he must use resources of other providers (and, in case, of which provider, or the “free” ones, made available by IaaS providers, and in what quantity);
- Which slices he should create (and use);
- What service to provide, and in what quantity;
- The security levels.

## The mathematical model

In this section, we describe the theoretical mathematical model previously mentioned. Let us introduce the first set of variables of the model.

Let $x_{gsp}\ge 0$ be the flow of service *g*, $g=1,\dots ,G$, provided by slice *s*, $s=1,\dots ,S$, of provider $p=1,\dots ,P$. We group these quantities, for all $s=1,\dots ,S$ and for all $p=1,\dots ,P$, into the vector $X_g\in {\mathbb {R}}_+^{SP}$. In turn, we group these quantities into the vector $X\in {\mathbb {R}}_+^{GSP}$. We denote by $y_{s{\tilde{p}}rp}\ge 0$ the flow of resource *r*, $r=1,\dots ,R$, from provider *p*, $p=1,\dots ,P+1$, to the slice *s*, $s=1,\dots ,S$, of provider ${\tilde{p}}$, ${\tilde{p}}=1,\dots ,P$. We observe that with $y_{s{\tilde{p}}r(P+1)}$ we indicate the flow of “free” resource *r* provided to slice *s* of provider ${\tilde{p}}$. We group these quantities, for all $s=1,\dots ,S$, ${\tilde{p}}=1,\dots ,P$, $r=1,\dots ,R$, $p=1,\dots ,P+1$, into the vector $Y\in {\mathbb {R}}_+^{SPR(P+1)}$. The parameters of the model are reported in Table 1.

**Table 1:** Parameters for the model

Notation	Parameters
$D_g$	The request for service *g*, $g=1,\dots ,G$. We assume such requests as fixed and known.
$D_{gp}$	The request of exclusive customers for service *g* of provider *p*, $g=1,\dots ,G$, $p=1,\dots ,P$.
$A_{rp}$	The quantity of available resource *r* owned by provider *p*, $r=1,\dots ,R$, $p=1,\dots ,P+1$. We observe that the quantity $A_{r(P+1)}$ represents the amount of ‘free” resource *r*.
${\overline{C}}_{s{\tilde{p}}}$	The maximum capacity of slice *s* of provider ${\tilde{p}}$, $s=1,\dots ,S$, ${\tilde{p}}=1,\dots ,P$.
$\gamma _{rg}$	The quantity of resource *r* needed to execute a unit of service *g*, $r=1,\dots ,R$, $g=1,\dots ,G$.
$B_{{\tilde{p}}}$	The limited budget of provider ${\tilde{p}}$ for cybersecurity investment, ${\tilde{p}}=1,\dots ,P$.

We now introduce the cost functions associated with transport/transmission of services, the rental of resources at the resource level and utilization/execution of resources and slices. We denote by:

- $c_{{\tilde{p}}}$ the total transport/transmission costs associated with service and resource flows for provider ${\tilde{p}}$. We suppose that such costs are defined as follows:
  $$\begin{aligned}\begin{aligned} c_{{\tilde{p}}}(X,Y)=&\sum _{s=1}^S\sum _{g=1}^Gc_{gs{\tilde{p}}}(x_{gs{\tilde{p}}})\\&+\sum _{r=1}^R\sum _{p=1}^{P+1}\sum _{s=1}^Sc_{s{\tilde{p}}rp}(y_{s{\tilde{p}}rp}),\quad \forall {\tilde{p}},\end{aligned} \end{aligned}$$
  where the first term of the above expression represents the total transmission/transport costs of services and the second one represents the total transmission/transport costs of resources. Particularly, we indicate with $c_{gs{\tilde{p}}}$ the cost to transmit the service *g* from slice *s* of the provider ${\tilde{p}}$ and we suppose it is a function of the flow $x_{gs{\tilde{p}}}$, namely
  $$\begin{aligned}\begin{aligned} c_{gs{\tilde{p}}}:=c_{gs{\tilde{p}}}(x_{gs{\tilde{p}}}),\quad \forall g,\forall s,\ \forall {\tilde{p}}\end{aligned} \end{aligned}$$
  and we indicate with $c_{s{\tilde{p}}rp}$ the cost to transmit or transport of resource *r* from provider *p* to slice *s* of the provider ${\tilde{p}}$. As before, we suppose that such functions depend on the flow $y_{s{\tilde{p}}rp}$, namely
  $$\begin{aligned} c_{s{\tilde{p}}rp}:=c_{s{\tilde{p}}rp}(y_{s{\tilde{p}}rp}),\quad \forall s,\ \forall {\tilde{p}},\ \forall r,\ \forall p.\end{aligned}$$
  We also suppose that, for all *p*, if service *g* does not use slice *s*, that is service *g* cannot be executed in slice *s*, the cost $c_{gsp}$ assumes a very high value ${\overline{M}}$, i.e. $c_{gsp}(x_{gsp})={\overline{M}}$.
- $c_{{\tilde{p}}}^{(A)}$ the total rental costs. We suppose that such costs are defined as follows:
  $$\begin{aligned}\begin{aligned} c_{{\tilde{p}}}^{(A)}(Y)=&\sum _{r=1}^R\sum _{p=1}^{(P+1)}\sum _{s=1}^Sc^{(A)}_{s{\tilde{p}}rp}(y_{s{\tilde{p}}rp})\\ -&\sum _{s=1}^S\sum _{p=1}^P\sum _{r=1}^Rc^{(A)}_{spr{\tilde{p}}}(y_{spr{\tilde{p}}}),\quad \forall {\tilde{p}},\end{aligned} \end{aligned}$$

**Fig. 2:**
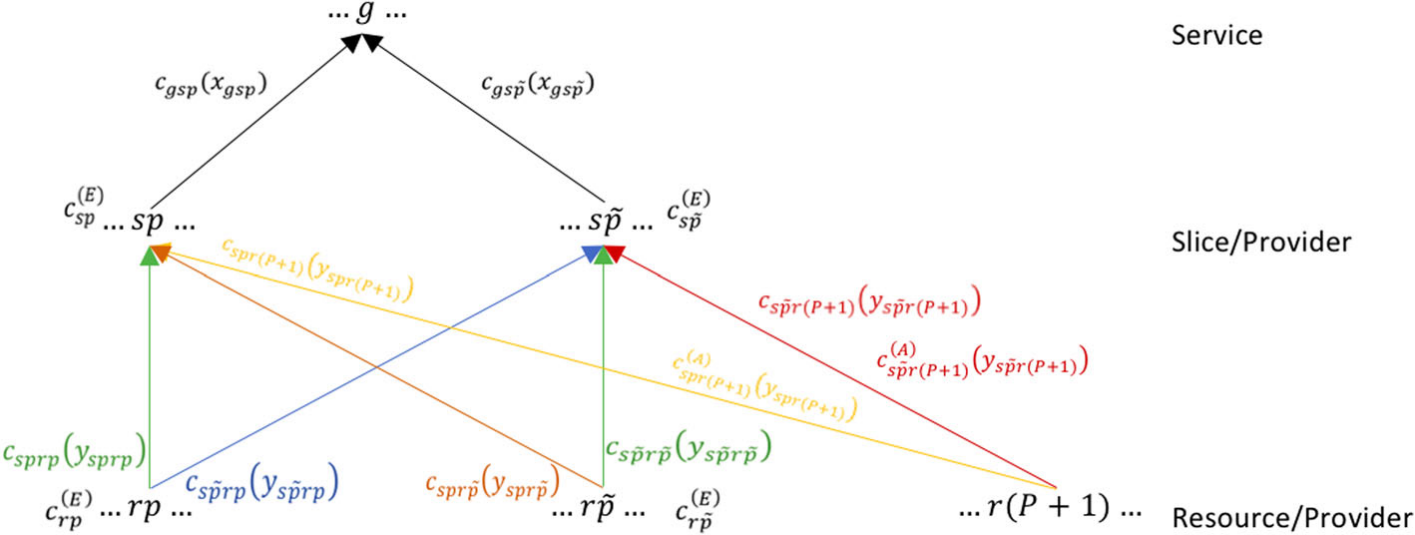
Detailed representation of the network: variables, transmission and rental cost functions

where the first term of the previous expression represents the total rental costs for all slices of provider ${\tilde{p}}$ to rent resources from the other services providers or from the IaaS providers and the second term represents the total revenue obtained by ${\tilde{p}}$ from leasing its resources to all slices of other providers and where we have supposed that

$$\begin{aligned} c^{(A)}_{s{\tilde{p}}rp}:=c^{(A)}_{s{\tilde{p}}rp}(y_{s{\tilde{p}}rp}),\quad \forall s,\ \forall {\tilde{p}},\ \forall r,\ \forall p, \end{aligned}$$

and

$$\begin{aligned} c^{(A)}_{spr{\tilde{p}}}:=c^{(A)}_{spr{\tilde{p}}}(y_{spr{\tilde{p}}}),\quad \forall s,\ \forall p,\ \forall r,\ \forall {\tilde{p}}. \end{aligned}$$

Moreover, we suppose that:

$$\begin{aligned} c^{(A)}_{s{\tilde{p}}r{\tilde{p}}}=0, \forall s,\ \forall r,\ \forall {\tilde{p}}, \end{aligned}$$

that is there is no cost or revenue from the rent for transactions between resources and slices of the same provider.

- $c_{{\tilde{p}}}^{(E)}$ the total utilization/execution costs. We suppose that such costs are defined as:
  $$\begin{aligned} \begin{aligned} c_{{\tilde{p}}}^{(E)}(X,Y)=&\sum _{r=1}^Rc^{(E)}_{r{\tilde{p}}}\left( \sum _{s=1}^S\sum _{p=1}^Py_{spr{\tilde{p}}}\right) \\&+\sum _{s=1}^Sc^{(E)}_{s{\tilde{p}}}\left( \sum _{g=1}^Gx_{gs{\tilde{p}}}\right) ,\quad \forall {\tilde{p}}, \end{aligned} \end{aligned}$$
  where the first term of the above expression represents the total utilization/execution costs incurred by provider ${\tilde{p}}$ to use/execute all his resources (used for himself or rented to other providers’ slices) and the second term represents the total utilization (execution) costs incurred by provider ${\tilde{p}}$ to use/execute all of his slices to run all the services provided to users.

Particularly, we suppose $c_{r{\tilde{p}}}^{(E)}$ as a function of the total flow of the resource *r* (belonging to provider ${\tilde{p}}$) used for all slices of all providers, that is $\displaystyle \sum _{s=1}^S\sum _{p=1}^Py_{spr{\tilde{p}}}$ and $c_{s{\tilde{p}}}^{(E)}$ as a function of the total flow of all services provided by the slice *s* of provider ${\tilde{p}}$, that is $\displaystyle \sum _{g=1}^Gx_{gs{\tilde{p}}}$.

As previously discussed, a fundamental aspect of 5G network architectures is security. Particularly, in this paper we suppose that the security level of each provider ${\tilde{p}}$, ${\tilde{p}}=1,\dots ,P$ depends on security levels in its resources nodes, in the links between all resources nodes and its slices, in its slice nodes and in the links between its slices and clients at the service level. Let us introduce the security variables. We denote by:

- $\sigma _{r{\tilde{p}}}\in [0;{\overline{\sigma }}_{r{\tilde{p}}}]$ the security level in resource node *r* of provider ${\tilde{p}}$, for all $r=1,\dots ,R$ and ${\tilde{p}}=1,\dots ,P$;
- $\sigma _{s{\tilde{p}}rp}\in [0;{\overline{\sigma }}_{s{\tilde{p}}rp}]$ the security level in the link connecting the resource node *r*, of provider *p* and slice *s* of provider ${\tilde{p}}$, $s=1,\dots ,S$, ${\tilde{p}}=1,\dots ,P$, $r=1,\dots ,R$, $p=1,\dots ,P+1$. As usual in this article, the variable $\sigma _{s{\tilde{p}}r(P+1)}$ indicates the security level in the link between the “free” resource node and slice *s* of provider ${\tilde{p}}$;
- $\sigma _{s{\tilde{p}}}\in [0;{\overline{\sigma }}_{s{\tilde{p}}}]$ the security level in the slice node *s* of provider ${\tilde{p}}$, $s=1,\dots ,S$; ${\tilde{p}}=1,\dots ,P$;
- $\sigma _{gs{\tilde{p}}}\in [0;{\overline{\sigma }}_{gs{\tilde{p}}}]$ the security level in the link between slice *s* of provider ${\tilde{p}}$ and costumers requiring service *g* at the service level, $g=1,\dots ,G$; $s=1,\dots ,S$; ${\tilde{p}}=1,\dots ,P$.

**Fig. 3:**
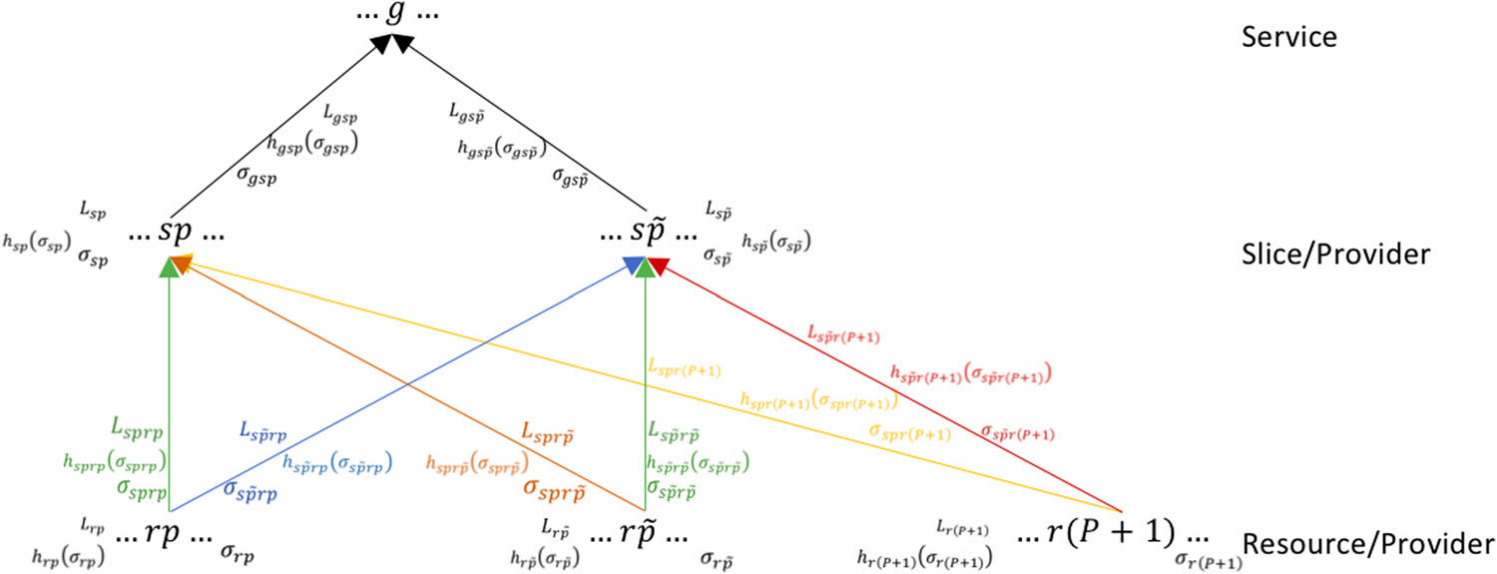
Detailed representation of the network: security levels, investment cost functions and financial damages

For a better comprehension of the variables, cost functions, investment cost functions and security levels, refer to Figs. 2 and 3.

The upper bounds ${\overline{\sigma }}_{r{\tilde{p}}},\ {\overline{\sigma }}_{s{\tilde{p}}rp},\ {\overline{\sigma }}_{s{\tilde{p}}},\ {\overline{\sigma }}_{gs{\tilde{p}}}<1$ exclude the unreal case in which suppliers reach a security level of 100%. We denote by $\sigma _{{\tilde{p}}}$ the security level for provider ${\tilde{p}}$, ${\tilde{p}}=1,\dots ,P$ and assume that it is given by the weighted average of the security levels introduced above:

1 $$\begin{aligned} \displaystyle \sigma _{{\tilde{p}}}= \frac{\Gamma ^N _{{\tilde{p}}}}{\Gamma ^D_{{\tilde{p}}}},\quad \forall {\tilde{p}}, \end{aligned}$$

where

$$\begin{aligned}\begin{aligned}&\Gamma ^N_{{\tilde{p}}}=\displaystyle \alpha ^{(1)}_{{\tilde{p}}}\sum _{r=1}^R\sigma _{r{\tilde{p}}} \left( \sum _{s=1}^S y_{s{\tilde{p}}r{\tilde{p}}}\right) +\alpha ^{(2)}_{{\tilde{p}}}\sum _{s=1}^S\sum _{r=1}^R\sum _{p=1}^{P+1}\sigma _{s{\tilde{p}}rp} y_{s{\tilde{p}}rp}\\&+\alpha ^{(3)}_{{\tilde{p}}}\sum _{s=1}^S\sigma _{s{\tilde{p}}} \left( \sum _{g=1}^G x_{gs{\tilde{p}}}\right) + \alpha ^{(4)}_{{\tilde{p}}}\sum _{g=1}^G\sum _{s=1}^S\sigma _{gs{\tilde{p}}}x_{gs{\tilde{p}}},\quad \forall {\tilde{p}}\end{aligned} \end{aligned}$$

and

$$\begin{aligned} \Gamma ^D_{{\tilde{p}}}=&\displaystyle \alpha ^{(1)}_{{\tilde{p}}}\sum _{r=1}^R \sum _{s=1}^S y_{s{\tilde{p}}r{\tilde{p}}} + \alpha ^{(2)}_{{\tilde{p}}}\sum _{s=1}^S\sum _{r=1}^R\sum _{p=1}^{P+1} y_{s{\tilde{p}}rp}\\&\, +\, \alpha ^{(3)}_{{\tilde{p}}}\sum \limits _{s=1}^S\sum \limits _{g=1}^G x_{gs{\tilde{p}}} + \alpha ^{(4)}_{{\tilde{p}}}\sum \limits _{g=1}^G\sum \limits _{s=1}^Sx_{gs{\tilde{p}}} \end{aligned}$$

where $\alpha ^{(1)}_{{\tilde{p}}}, \alpha ^{(2)}_{{\tilde{p}}}, \alpha ^{(3)}_{{\tilde{p}}}$ and $\alpha ^{(4)}_{{\tilde{p}}}\ge 0$ denote the weights, established by the provider ${\tilde{p}}$, ${\tilde{p}}=1,\dots ,P$, associated with the security levels in the resources nodes, in the links resources-slices, in the slice nodes and in the links slices-services, respectively. Moreover, we suppose that $\alpha ^{(1)}_{{\tilde{p}}}+ \alpha ^{(2)}_{{\tilde{p}}}+ \alpha ^{(3)}_{{\tilde{p}}}+\alpha ^{(4)}_{{\tilde{p}}}=1.$ Particularly, $\Gamma _{{\tilde{p}}}^D$ represents the $\alpha _{{\tilde{p}}}$-weighted sum of performed activities (in resource nodes, in resource-slice links, in slice nodes and in slice-service links) of provider ${\tilde{p}}$ while $\Gamma _{{\tilde{p}}}^N$ represents the $\sigma _{{\tilde{p}}}$-weighted $\Gamma _{{\tilde{p}}}^D$. Note that the presence of the aforementioned weights reflects the preference of each provider regarding organizational, management or economic issues.

As previously studied in existing literature (see, for instance, Colajanni et al. (2018), Colajanni et al. (2020), Nagurney et al. (2017) and Nagurney and Shukla (2017)), each provider can increase its security levels by incurring investment costs, defined as follows:

$$\begin{aligned} h_{r{\tilde{p}}}(\sigma _{r{\tilde{p}}})= & {} \beta _{r{\tilde{p}}}\left( \frac{1}{\sqrt{(1-\sigma _{r{\tilde{p}}})}}-1 \right) ,\quad \forall r,\ \forall {\tilde{p}}, \\ h_{s{\tilde{p}}rp}(\sigma _{s{\tilde{p}}rp})= & {} \beta _{s{\tilde{p}}rp}\left( \frac{1}{\sqrt{(1-\sigma _{s{\tilde{p}}rp})}}-1 \right) ,\quad \forall s,\ \forall {\tilde{p}},\ \forall r,\ \forall p \\ h_{s{\tilde{p}}}(\sigma _{s{\tilde{p}}})= & {} \beta '_{s{\tilde{p}}}\left( \frac{1}{\sqrt{(1-\sigma _{s{\tilde{p}}})}}-1 \right) ,\quad \forall s,\ \forall {\tilde{p}}; \\ h_{gs{\tilde{p}}}(\sigma _{gs{\tilde{p}}})= & {} \beta _{gs{\tilde{p}}}\left( \frac{1}{\sqrt{(1-\sigma _{gs{\tilde{p}}})}}-1 \right) ,\quad \forall g,\ \forall s,\ \forall {\tilde{p}}. \end{aligned}$$

We observe that the above investment costs functions are well-defined since we have assumed that ${\overline{\sigma }}_{r{\tilde{p}}},$ ${\overline{\sigma }}_{s{\tilde{p}}rp},$ ${\overline{\sigma }}_{s{\tilde{p}}},$ ${\overline{\sigma }}_{gs{\tilde{p}}}<1$ and increasing functions with respect to its own variable.

The probability of a successful cyber-attack on a node or on a link of the network is equivalent to the corresponding level of vulnerability of such node or link. Hence, for instance in the case of the resource *r* of provider ${\tilde{p}}$, this probability is $(1-\sigma _{r{\tilde{p}}})$, and it depends on the security level $\sigma _{r{\tilde{p}}}$, $r=1,\dots ,R, {\tilde{p}}=1,\dots ,P$.

Such a probability is independent on the probability $\Psi _{r{\tilde{p}}}$ that the resource *r* of provider ${\tilde{p}}$ is attacked. Therefore, the probability of a successful cyberattack on resource node *r* of provider ${\tilde{p}}$ can be expressed by the product of the two probabilities: $\Psi _{r{\tilde{p}}}\cdot (1-\sigma _{r{\tilde{p}}})$. The probability $\Psi _{r{\tilde{p}}}$ is determined, in turn, by the product of $\psi$, which represents the probability that the whole network is attacked, the conditional probability, $\psi _{{\tilde{p}}}$, that provider ${\tilde{p}}$ suffers an attack, assuming that the network is attacked and the conditional probability, $\psi _{r{\tilde{p}}}$ that the resource node *r* of provider ${\tilde{p}}$ suffers an attack, assuming that the provider ${\tilde{p}}$ is attacked, that is: $\Psi _{r{\tilde{p}}}=\psi \cdot \psi _{{\tilde{p}}}\cdot \psi _{r{\tilde{p}}}$. It trivially follows from the definition of probability of intersection between events.

In this paper, the probability $\psi$ that the whole network suffers a cyberattack is considered fixed and known, as determined by factors external to the network.

The conditional probability $\psi _{{\tilde{p}}}$ that provider ${\tilde{p}}$ is attacked, assuming that the whole network suffers a cyberattack, depends on the activity of provider ${\tilde{p}}$ with respect to the total activity of the entire network and, therefore, with respect to the activity of all providers of the network. Hence, it can be defined as follows:

$$\begin{aligned} \psi _{{\tilde{p}}}=\frac{\displaystyle \Lambda ^N_{{\tilde{p}}}}{\displaystyle \Lambda ^D},\quad \forall {\tilde{p}}, \end{aligned}$$

where

$$\begin{aligned} \Lambda ^N_{{\tilde{p}}}= & {} \displaystyle \sum _{r=1}^R\left( \sum _{p=1}^P \sum _{s=1}^S y_{spr{\tilde{p}}} \right) + \sum _{s=1}^S\sum _{r=1}^R\sum _{p=1}^{P+1} y_{s{\tilde{p}}rp} \\&+ \sum _{s=1}^S\left( \sum _{g=1}^G x_{gs{\tilde{p}}}\right) + \sum _{g=1}^G\sum _{s=1}^Sx_{gs{\tilde{p}}}, \quad \forall {\tilde{p}} \end{aligned}$$

and

$$\begin{aligned} \Lambda ^D= & {} \displaystyle \sum _{{\tilde{p}}}^P\Lambda ^N_{{\tilde{p}}}= \sum _{{\tilde{p}}=1}^P\left[ \sum _{r=1}^R\left( \sum _{p=1}^P \sum _{s=1}^S y_{spr{\tilde{p}}} \right) \right. \\&+ \sum _{s=1}^S\sum _{r=1}^R\sum _{p=1}^{P+1} y_{s{\tilde{p}}rp} +\left. \sum _{s=1}^S\left( \sum _{g=1}^G x_{gs{\tilde{p}}}\right) + \sum _{g=1}^G\sum _{s=1}^Sx_{gs{\tilde{p}}}\right] . \end{aligned}$$

Finally, the conditional probability $\psi _{r{\tilde{p}}}$ that the resource node *r* of provider ${\tilde{p}}$ suffers a cyberattack, assuming that the provider ${\tilde{p}}$ is attacked, depends on the activity of resource *r* with respect to the total activities of the provider ${\tilde{p}}$. Hence, if a resource is unused, the probability that it is attacked is null. If, on the contrary, a resource of the network is used intensively, the probability of an attack is greater. Therefore, the expression of $\psi _{r{\tilde{p}}}$ reads as follows:

$$\begin{aligned} \displaystyle \psi _{r{\tilde{p}}}=\frac{\displaystyle \sum _{p=1}^P \sum _{s=1}^S y_{spr{\tilde{p}}} }{\Lambda ^N_{{\tilde{p}}}},\quad \forall r,\ \forall {\tilde{p}}. \end{aligned}$$

In conclusion, we have:

$$\begin{aligned} \Psi _{r{\tilde{p}}}= & {} \psi \cdot \psi _{{\tilde{p}}}\cdot \psi _{r{\tilde{p}}}= \frac{\psi \displaystyle \sum _{p=1}^P \sum _{s=1}^S y_{spr{\tilde{p}}} }{\displaystyle \Lambda ^D},\forall r,\ \forall {\tilde{p}}. \end{aligned}$$

If a cyberattack is successful, the provider of the network suffers a damage. In the event of a successful cyberattack on the resource node *r* of provider ${\tilde{p}}$, we denote by $L_{r{\tilde{p}}}$ the damage to be paid per unit of attacked resource used by some slices. Therefore, the expected financial damage in the case of a successful cyberattack on the resource *r* of provider ${\tilde{p}}$ is given by:

$$\begin{aligned} \psi \cdot \psi _{{\tilde{p}}}\cdot \psi _{r{\tilde{p}}}\cdot (1-\sigma _{r{\tilde{p}}})\cdot L_{r{\tilde{p}}}\cdot \left( \sum _{p=1}^P\sum _{s=1}^Sy_{spr{\tilde{p}}}\right) . \end{aligned}$$

Note that, unlike the analysis of the security level, the expected financial damage also takes into account the amount of resource rented to other providers, since in this case it is necessary to pay the damage suffered by the providers to which the same resource is not guaranteed. On the other hand, it must be taken into account that provider ${\tilde{p}}$ obtains from the other providers the value of the possible damage in case of success of the attack to the resources rented to him. Therefore, provider ${\tilde{p}}$ obtains the following quantity:

$$\begin{aligned} \sum _{r=1}^R\sum _ {\begin{array}{c} {p=1}\\ {p\ne {\tilde{p}}} \end{array}} ^{P+1}\psi \cdot \psi _{{p}}\cdot \psi _{r{p}}\cdot (1-\sigma _{r{p}})\cdot L_{r{p}}\cdot \left( \sum _{s=1}^Sy_{s{\tilde{p}}rp}\right) . \end{aligned}$$

Similar considerations can be made for the other security levels, for which, therefore, we have:

$$\begin{aligned} \displaystyle \psi _{s{\tilde{p}}rp}=\frac{\displaystyle y_{s{\tilde{p}}rp}}{\displaystyle \Lambda ^N_{{\tilde{p}}}},\quad \forall s, \forall {\tilde{p}},\ \forall r,\ \forall p,\\\displaystyle \psi _{s{\tilde{p}}}=\frac{\displaystyle \sum _{g=1}^G x_{gs{\tilde{p}}}}{\displaystyle \Lambda ^N_{{\tilde{p}}}},\quad \forall s,\ \forall {\tilde{p}} \end{aligned}$$

and

$$\begin{aligned} \displaystyle \psi _{gs{\tilde{p}}}=\frac{\displaystyle x_{gs{\tilde{p}}}}{\displaystyle \Lambda ^N_{{\tilde{p}}}},\quad \forall g,\ \forall s,\ \forall {\tilde{p}}. \end{aligned}$$

Moreover, we observe that, since the objective function is summed with respect to ${\tilde{p}}$, the sum of the damages paid by a provider ${\tilde{p}}$ and the damages received by all the other providers from ${\tilde{p}}$ are null. Hence, the following term in the objective function:

$$\begin{aligned}&\sum \limits _{{\tilde{p}}=1}^P\Bigg \{-\sum \limits _{r=1}^R \psi \cdot \psi _{{\tilde{p}}}\cdot \psi _{r{\tilde{p}}}\cdot (1-\sigma _{r{\tilde{p}}})\cdot L_{r{\tilde{p}}}\cdot \left( \sum \limits _{p=1}^P\sum \limits _{s=1}^Sy_{spr{\tilde{p}}}\right) \\&+\sum \limits _{r=1}^R\sum \limits _ {\begin{array}{c} {p=1}\\ {p\ne {\tilde{p}}} \end{array}} ^{P+1}\psi \cdot \psi _{{p}}\cdot \psi _{r{p}}\cdot (1-\sigma _{r{p}})\cdot L_{r{p}}\cdot \left( \sum \limits _{s=1}^Sy_{s{\tilde{p}}rp}\right) \Bigg \}, \end{aligned}$$

becomes:

$$\begin{aligned}&\sum \limits _{{\tilde{p}}=1}^P\left\{ -\sum \limits _{r=1}^R \psi \cdot \psi _{{\tilde{p}}}\cdot \psi _{r{\tilde{p}}}\cdot (1-\sigma _{r{\tilde{p}}})\cdot L_{r{\tilde{p}}}\cdot \left( \sum \limits _{s=1}^Sy_{s{\tilde{p}}r{\tilde{p}}}\right) \right. \\&+\sum \limits _{r=1}^R\psi \cdot \psi _{{(P+1)}}\cdot \psi _{r{(P+1)}}\cdot (1-\sigma _{r{(P+1)}})\\&\left. \qquad \cdot L_{r{(P+1)}}\cdot \left( \sum \limits _{s=1}^Sy_{s{\tilde{p}}r(P+1)}\right) \right\} . \end{aligned}$$

Likewise, all the rental costs and rental revenues are null, except the costs to rent free resources:

$$\begin{aligned}&\sum \limits _{{\tilde{p}}=1}^P\left\{ -\sum \limits _{r=1}^R\sum \limits _{p=1}^{(P+1)}\sum \limits _{s=1}^Sc^{(A)}_{s{\tilde{p}}rp}(y_{s{\tilde{p}}rp})\right. \\&\left. \qquad +\sum \limits _{s=1}^S\sum \limits _{p=1}^P\sum \limits _{r=1}^Rc^{(A)}_{spr{\tilde{p}}}(y_{spr{\tilde{p}}})\right\} \\&\quad =\sum \limits _{{\tilde{p}}=1}^P\left\{ -\sum \limits _{r=1}^R\sum _{s=1}^Sc^{(A)}_{s{\tilde{p}}r(P+1)}(y_{s{\tilde{p}}r(P+1)})\right\} . \end{aligned}$$

In this paper, we have supposed that the probabilities of cyberattack on nodes or links, per unit of executed activity, i.e. used resource or executed service for nodes and transmitted flow for links, are equivalent. However, it is easy to generalize the model to the case in which these probabilities are different, multiplying $\psi _{r{\tilde{p}}}$, $\psi _{s{\tilde{p}}rp}$, $\psi _{s{\tilde{p}}}$ and $\psi _{gs{\tilde{p}}}$ by appropriate weights.

Finally, we denote by $\rho _{g{\tilde{p}}}$, for all $g=1,\dots ,G$ and ${\tilde{p}}=1,\dots ,P$, the revenue of provider ${\tilde{p}}$ obtained by the sale of service *g* and we suppose $\rho _{g{\tilde{p}}}$ as a function of the vector of service flows and the security level of provider ${\tilde{p}}$, that is:An analytic expression for the revenue of provider ${\tilde{p}}$ is provided by equation (2):2where $\alpha _{g{\tilde{p}}}>0$ enables distinct providers to have different revenue functions based on their size and their needs. It is straightforward to verify that $\rho _{g{\tilde{p}}}$ is a decreasing function with respect to $X_g$ and this reflects the idea according to which a higher revenue is obtained for services that are difficult to fulfil. Particularly, for each service *g*, $g=1,\dots ,G$, when the total amount of performed service equals the demand $D_g$ for that service, the unit revenue for each provider ${\tilde{p}}$, ${\tilde{p}}=1,\dots ,P$, reaches the value $\rho _{min}$, assumed fixed and the same for all the providers of the network.

As previously mentioned, we want to provide a system-optimization perspective for the entire supply chain network, analysing the system from the point of view of the network as well as service providers. Hence, the objective is to determine the optimal flows between the network layers (also consisting of UAVs supported and connected by 5G technology) that maximize the objective function consisting of the profit of all providers, given by the difference between the total revenue obtained from the sale of 5G services and the rental of resources and the total transmission/transport, rental and utilization or execution costs. Moreover, we also want to determine the optimal security levels of the network’s providers which minimize the expected financial damage in case of successful cyberattacks.

The formulation of the problem reads as follows:

$$\begin{aligned}&\max \sum \limits _{{\tilde{p}}=1}^P\Bigg \{\sum \limits _{g=1}^G\rho _{g{\tilde{p}}}(X_{g},\sigma _{{\tilde{p}}}) -\sum \limits _{s=1}^S\sum _{g=1}^Gc_{gs{\tilde{p}}}(x_{gs{\tilde{p}}})\\&\quad -\sum \limits {r=1}^R\sum _{p=1}^{P+1}\sum \limits _{s=1}^Sc_{s{\tilde{p}}rp}(y_{s{\tilde{p}}rp})-\sum \limits _{r=1}^R\sum \limits _{s=1}^Sc^{(A)}_{s{\tilde{p}}r(P+1)}(y_{s{\tilde{p}}r(P+1)})\\&\quad -\sum \limits _{r=1}^Rc^{(E)}_{r{\tilde{p}}}\left( \sum \limits _{s=1}^S\sum _{p=1}^Py_{spr{\tilde{p}}}\right) -\sum \limits _{s=1}^Sc^{(E)}_{s{\tilde{p}}}\left( \sum \limits _{g=1}^Gx_{gs{\tilde{p}}}\right) \\&\quad -\left[ \sum \limits _{r=1}^R h_{r{\tilde{p}}}(\sigma _{r{\tilde{p}}})+ \sum \limits _{s=1}^S\sum _{r=1}^R\sum \limits _{p=1}^{P+1} h_{s{\tilde{p}}rp}(\sigma _{s{\tilde{p}}rp})+ \sum \limits _{s=1}^S h_{s{\tilde{p}}}(\sigma _{s{\tilde{p}}})\right. \\&\quad \left. + \sum \limits _{g=1}^G\sum _{s=1}^S h_{gs{\tilde{p}}}(\sigma _{gs{\tilde{p}}})\right] \\&\quad -\sum \limits _{r=1}^R \psi \cdot \psi _{{\tilde{p}}}\cdot \psi _{r{\tilde{p}}}\cdot (1-\sigma _{r{\tilde{p}}})\cdot L_{r{\tilde{p}}}\cdot \left( \sum \limits _{s=1}^Sy_{s{\tilde{p}}r{\tilde{p}}}\right) \\&\quad +\sum \limits _{r=1}^R\psi \cdot \psi _{{(P+1)}}\cdot \psi _{r{(P+1)}}\cdot (1-\sigma _{r{(P+1)}})\\&\quad \cdot L_{r{(P+1)}}\cdot \left( \sum \limits _{s=1}^Sy_{s{\tilde{p}}r(P+1)}\right) \\&\quad -\left[ \sum \limits _{s=1}^S\sum \limits _{r=1}^R\sum _{p=1}^{P+1} \psi \cdot \psi _{{\tilde{p}}}\cdot \psi _{s{\tilde{p}}rp}\cdot (1-\sigma _{s{\tilde{p}}rp})\cdot L_{s{\tilde{p}}rp}\cdot y_{s{\tilde{p}}rp}\right. \\&\quad \left. +\sum \limits _{s=1}^S \psi \cdot \psi _{{\tilde{p}}}\cdot \psi _{s{\tilde{p}}}\cdot (1-\sigma _{s{\tilde{p}}})\cdot L_{s{\tilde{p}}}\cdot \left( \sum \limits _{g=1}^Gx_{gs{\tilde{p}}}\right) \right. \end{aligned}$$

3 $$\begin{aligned} \quad +\left. \sum \limits _{g=1}^G\sum \limits _{s=1}^S \psi \cdot \psi _{{\tilde{p}}}\cdot \psi _{gs{\tilde{p}}}\cdot (1-\sigma _{gs{\tilde{p}}})\cdot L_{gs{\tilde{p}}}\cdot x_{gs{\tilde{p}}}\right] \Bigg \},\end{aligned}$$

subject to:

4 $$\begin{aligned}&\sum \limits _{s=1}^S\sum \limits _{p=1}^Px_{gsp}\le D_{g}, \quad \forall g,\end{aligned}$$

5 $$\begin{aligned}&\sum \limits _{s=1}^Sx_{gsp}\ge D_{gp}, \quad \forall g, \forall p,\end{aligned}$$

6 $$\begin{aligned}&\sum \limits _{s=1}^S\sum \limits _{{\tilde{p}}=1}^{P}y_{s{\tilde{p}}rp}\le A_{rp}, \quad \forall r, \forall {p}=1,...,P+1,\end{aligned}$$

7 $$\begin{aligned}&\sum \limits _{r=1}^R\sum \limits _{p=1}^{P+1}y_{s{\tilde{p}}rp}\le {\overline{C}}_{s{\tilde{p}}}, \quad \forall s, \forall {\tilde{p}},\end{aligned}$$

8 $$\begin{aligned}&\sum \limits _{p=1}^{P+1} y_{s{\tilde{p}}rp}\ge \sum \limits _{g=1}^G\gamma _{rg}x_{gs{\tilde{p}}},\quad \forall r, \forall s, \forall {\tilde{p}},\end{aligned}$$

9 $$\begin{aligned}&\begin{aligned}&\displaystyle \sum _{r=1}^R \sum _{s=1}^S y_{s{\tilde{p}}r{\tilde{p}}} + \sum _{s=1}^S\sum _{r=1}^R\sum _{p=1}^{P+1} y_{s{\tilde{p}}rp} + \sum _{s=1}^S\sum _{g=1}^G x_{gs{\tilde{p}}}\\&+\sum _{g=1}^G\sum _{s=1}^Sx_{gs{\tilde{p}}}>0,\quad \forall {\tilde{p}}, \end{aligned} \end{aligned}$$

10 $$\begin{aligned}&\begin{aligned}&\sum _{r=1}^R h_{r{\tilde{p}}}(\sigma _{r{\tilde{p}}})+ \sum _{s=1}^S\sum _{r=1}^R\sum _{p=1}^{P+1} h_{s{\tilde{p}}rp}(\sigma _{s{\tilde{p}}rp})\\ {}&+ \sum _{s=1}^S h_{s{\tilde{p}}}(\sigma _{s{\tilde{p}}})+ \sum _{g=1}^G\sum _{s=1}^S h_{gs{\tilde{p}}}(\sigma _{gs{\tilde{p}}})\le B_{{\tilde{p}}},\quad \forall {\tilde{p}}. \end{aligned} \end{aligned}$$

11 $$\begin{aligned}&\begin{aligned}&x_{gsp},\ y_{s{\tilde{p}}rp}\ge 0,\\&\sigma _{r{\tilde{p}}}\in [0,{\overline{\sigma }}_{r{\tilde{p}}}],\ \sigma _{s{\tilde{p}}rp}\in [0,{\overline{\sigma }}_{s{\tilde{p}}rp}],\ \sigma _{s{\tilde{p}}}\in [0,{\overline{\sigma }}_{s{\tilde{p}}}],\\&\sigma _{gs{\tilde{p}}}\in [0,{\overline{\sigma }}_{gs{\tilde{p}}}],\quad \forall s,\ \forall {\tilde{p}}, \forall g,\ \forall s, \forall p.\end{aligned} \end{aligned}$$

Constraint (4) states that, for each service *g*, no more service can be provided than the requested one.

Constraint (5) means that, for each service *g*, the demand of exclusive clients of each provider *p* must be satisfied.

Constraint (6) ensures that the amount of resource *r* that provider *p* transmits to all slices of all other providers does not exceed the amount of resource *r* owned by *p*.

Constraint (7) states that, for each provider ${\tilde{p}}$, the total amount of resources transmitted by all other providers to slice *s* of provider ${\tilde{p}}$ cannot exceed the maximum capacity of such a slice.

Constraint (8) ensures that, in each slice *s* of provider ${\tilde{p}}$, there are all the resources necessary to provide services. Thereby, if some resource is not sufficient for the execution of the service, this service is not provided.

In this paper, we are assuming that each provider ${\tilde{p}}$ of the network performs at least one function in the network and this feature is guaranteed by constraint (9). Moreover, the presence of this constraint ensures that all the conditional probabilities introduced above are well-defined, since their denominators are non-null.

Constraint (10) represents a nonlinear budget constraint for each provider ${\tilde{p}}$. It ensures that the sum of investment costs to increase the cybersecurity levels does not exceed the limited budget of provider. Finally, the latest constraint family defines the domain of the variables of the problem.

## A heuristic approach

In order to solve real instances, we modified the classical Genetic Algorithm (GA)-based method to find the optimal configuration of our non-linear constrained optimization problem, by optimizing the network’s providers profits and the expected financial damage in case of successful cyberattacks. The algorithm presented in this section is tested and calibrated in Subsection 4.1. A comparison is also carried out to evaluate the performance of the standard GA approach with our modified GA approach.

We consider the equivalent minimization problem of (3), namely:

$$\begin{aligned} \max {F (X,Y,\sigma )}=-\min -{F (X,Y,\sigma )} \end{aligned}$$

subject to (4)–(11).

For simplicity, we rename the feasible vector $(X,Y,\sigma )$ as *feavec*. The heuristic approach we propose consists of the following steps:

- **Step 1: Initial population generation** . We generate $dim\_pop$ feasible vectors as follows.
  - **Step 1.1**. We select the provider ${\tilde{p}}$ with the higher weighted sum of all the associated costs, that we call $c_{{\tilde{p}}}^{(w)}$. F or all the services ${\tilde{g}}$, the flow of service $x_{{\tilde{g}}{\tilde{s}}{\tilde{p}}}$ is set equal to the demand $D_{{\tilde{g}}{\tilde{p}}}$ increased by an error $\varepsilon _{{\tilde{g}}{\tilde{p}}}^{exp}$, exponentially distributed in $[0, D_{{\tilde{g}}}-\sum _{p=1}^PD_{{\tilde{g}}p}]$: $x_{{\tilde{g}}{\tilde{s}}{\tilde{p}}}=D_{{\tilde{g}}{\tilde{p}}}+\varepsilon _{{\tilde{g}}{\tilde{p}}}^{exp}.$ The other flows $x_{{\tilde{g}}{\tilde{s}}p}$, sorted in descending order of the associated weighted costs sum $c_{p}^{(w)}$, are obtained as $x_{{\tilde{g}}{\tilde{s}}p}=D_{{\tilde{g}}}-\sum _{p'\in {\tilde{P}}_p}x_{{\tilde{g}}{\tilde{s}}p'}-\varepsilon _{{\tilde{g}}{\tilde{s}}p}^r$, where ${\tilde{P}}_p=\{p'=1,\ldots ,P\;|\; c_{p'}^{(w)}>c_p^{(w)}\}$ and the error $\varepsilon _{{\tilde{g}}{\tilde{s}}p}^{rnd}$ is randomly uniformly distributed in $[0, D_{{\tilde{g}}}-\sum _{p'\in {\tilde{P}}_p}x_{{\tilde{g}}{\tilde{s}}p'}-D_{{\tilde{g}}p}]$.
  - **Step1.2**. For all the providers ${\tilde{p}}$, for all the slices ${\tilde{s}}$ and for all the resources ${\tilde{r}}$ in the network, we first select the provider $\bar{p}$ such that the cost $c_{{\tilde{s}}{\tilde{p}}{\tilde{r}}\bar{p}}=min_p\{c_{{\tilde{s}}{\tilde{p}}{\tilde{r}}p}\}$, and we set the flow $y_{{\tilde{s}}{\tilde{p}}{\tilde{r}}\bar{p}}=\gamma _{{\tilde{r}}{\tilde{g}}}\cdot x_{{\tilde{g}}{\tilde{s}}{\tilde{p}}}-\varepsilon _{{\tilde{s}}{\tilde{p}}{\tilde{r}}\bar{p}}^{exp}$, where the error $\varepsilon _{{\tilde{s}}{\tilde{p}}{\tilde{r}}\bar{p}}^{exp}$ is exponentially distributed in $[0, \gamma _{{\tilde{r}}{\tilde{g}}}\cdot x_{{\tilde{g}}{\tilde{s}}{\tilde{p}}}]$. The other flows $y_{{\tilde{s}}{\tilde{p}}{\tilde{r}}p}$, associated with the providers sorted in increasing order of the associated cost, are obtained as: $y_{{\tilde{s}}{\tilde{p}}{\tilde{r}}p}=\gamma _{{\tilde{r}}{\tilde{g}}}{\cdot } x_{{\tilde{g}}{\tilde{s}}{\tilde{p}}} - \sum _{\bar{p}\in \bar{P}_p} y_{{\tilde{s}}{\tilde{p}}{\tilde{r}}\bar{p}}- \varepsilon _{{\tilde{s}}{\tilde{p}}{\tilde{r}}p}^{rnd},$ where the error $\varepsilon _{{\tilde{s}}{\tilde{p}}{\tilde{r}}p}^{rnd}$ is uniformly distributed in $[0, \gamma _{{\tilde{r}}{\tilde{g}}\cdot x_{{\tilde{g}}{\tilde{s}}{\tilde{p}}} - \sum _{\bar{p}\in \bar{P}_p} y_{{\tilde{s}}{\tilde{p}}{\tilde{r}}\bar{p}} } ]$ and $\bar{P}_p=\{\bar{p}=1,\ldots ,P\; | \; c_{{\tilde{s}}{\tilde{p}}{\tilde{r}}\bar{p}} < c_{{\tilde{s}}{\tilde{p}}{\tilde{r}}p}\}$. The last flow associated with the cost $c_{{\tilde{s}}{\tilde{p}}{\tilde{r}}\bar{\bar{p}}}=max_p\{c_{{\tilde{s}}{\tilde{p}}{\tilde{r}}p}\}$ is obtained as $y_{{\tilde{s}}{\tilde{p}}{\tilde{r}}\bar{\bar{p}}}=\gamma _{{\tilde{r}}{\tilde{g}}}x_{{\tilde{g}}{\tilde{s}}\bar{\bar{p}}}-\sum \limits _{p\in \bar{\bar{P}}} y_{{\tilde{s}}{\tilde{p}}{\tilde{r}}p}$, where $\bar{\bar{P}}=\{p=1,\ldots ,P\;|\; c_{{\tilde{s}}{\tilde{p}}{\tilde{r}}p} < c_{{\tilde{s}}{\tilde{p}}{\tilde{r}}\bar{\bar{p}}}\}$.
  - **Step1.3**. Note that, for each provider ${\tilde{p}}$, each weight $\alpha _{{\tilde{p}}}^{(1)}$, $\alpha _{{\tilde{p}}}^{(2)}$, $\alpha _{{\tilde{p}}}^{(3)}$ and $\alpha _{{\tilde{p}}}^{(4)}$ is associated with some security variables (see (1), the security level for each provider ${\tilde{p}}$). Specifically, $\alpha _{{\tilde{p}}}^{(1)}$ is associated with the $\sigma _{r{\tilde{p}}}$ variables, $\alpha _{{\tilde{p}}}^{(2)}$ with $\sigma _{s{\tilde{p}}rp}$, $\alpha _{{\tilde{p}}}^{(3)}$ with $\sigma _{s{\tilde{p}}}$ and $\alpha _{{\tilde{p}}}^{(4)}$ with $\sigma _{gs{\tilde{p}}}$. We now consider these weights ($\alpha _{{\tilde{p}}}^{(1)}$, $\alpha _{{\tilde{p}}}^{(2)}$, $\alpha _{{\tilde{p}}}^{(3)}$, $\alpha _{{\tilde{p}}}^{(4)}$), and we sort them in ascending order. So, we first consider the $\sigma$ variable corresponding to the higher $\alpha$ and we define it as $\sigma = {\overline{\sigma }} - \varepsilon _{exp}$, where ${\overline{\sigma }}$ was the upper bound of $\sigma$ (see constraint (11) and the error $\varepsilon _{exp}$ varies with exponential distribution in $[0,{\overline{\sigma }}]$. To the $\sigma$ with the second higher $\alpha$ weight, we associate the value ${\overline{\sigma }} - \varepsilon _{exp}-\varepsilon _{exp}^{(2)}$, with $\varepsilon _{eps}^{(2)} \in [0,{\overline{\sigma }} - \varepsilon _{exp} ]$. The other $\sigma$ variables are defined as the difference between ${\overline{\sigma }}$ and a random exponential distributed error in [0;1].
  Afterwards, we check if all the generated vectors satisfy the (4)–(11) constraints. Then, we evaluate each vector, and we keep $\frac{1}{100}\cdot dim\_pop$ number of *feavec* vectors with the higher objective function value. We store all such vectors in a $\frac{1}{100}\cdot dim\_pop\times feavec-$ length matrix, which will constitute the initial population, denoted by ${\mathcal {P}}$.
- **Step 2. Selection**. For all the $\frac{1}{100}\cdot dim\_pop$ vectors in ${\mathcal {P}}$, we denote the generic one with $P_i$ $i=1,\dots , \frac{1}{100}\cdot dim\_pop$, and we associate with it the probability $p_i=\frac{F_{max}-F_i}{F_{max}-F_{min}} \in [0,1]$, where $F_{max}$ and $F_{min}$ are the highest and the lowest values of the objective function obtained from the population vectors, respectively, while $F_i$ is the value of objective function calculated in $P_i$. We consider that for each $P_i$ vector the cumulative probability distribution $prev\_prob_i$ is equal to the $prev\_prob_{i-1}$ of the previous vector in ${\mathcal {P}}$ (where $prev\_prob_{0}=0$) plus
  $$\begin{aligned}\frac{p_i}{\sum _{j}^{\frac{1}{100}\cdot dim\_pop}p_j}. \end{aligned}$$
  We select *nrand* values in [0, 1] uniformly distributed, and we denote the typical value by $vrand_j,\; \forall j=1,\ldots , nrand$. We include in the new “Parents selection” matrix the $i-th$ population vector, $P_i$, if *i* is the minimum index such that $vrand_j<prev\_prob_i$ $\forall j=1,..,nrand$. We remove the repeated vector. Note that the number of the selected vectors could be less than *nrand*. Observe that we could not use the classical Roulette Wheel Selection, because the objective functions (fitness) could assume positive or negative values.
- **Step 3. Crossover**. We generate $var\_cross$, a random integer values vector drawn from a uniform distribution in the close interval $[1,GSP+SPR(P+1)]$ and whose dimension is chosen randomly in $[2,GSP+SPR(P+1)]$. We remove the repeated value. Each of its component corresponds to one of the first GSP+SPR(P+1) *feavec* components, that are the service and resource flows, respectively (X,Y). For each service and/or resource flows of the vector selected, namely for each $var\_cross$ component, we sort the corresponding security variables $\sigma$ (following the same order of growth). Such correspondence between (*X*, *Y*) variables and $(\sigma )$ is obtained from the term of the objective function (3) related to the expected financial damage in case of successful cyberattack. We include to the population ${\mathcal {P}}$ only the feasible new vectors, which updated dimension $dim\_new\_pop$ could be grater than $dim\_pop$.
- **Step 4. Mutation**.
  - **Step 4.1**. In order to select the (X,Y) components of the population vectors, as in the previous Crossover Step, we generate $var\_mut$, a random integer values vector drawn from a uniform distribution in the close interval $[1,GSP+SPR(P+1)]$ and whose dimension is chosen randomly in $[1,GSP+SPR(P+1)]$. We remove the repeated value. We generate the vector $pop\_mut$, random integer values vector drawn from a uniform distribution in the close interval $[1,dim\_new\_pop]$ and whose length is $\lceil \frac{dim\_new\_pop}{10} \rceil$. We add to the $i-th$ variable, with $i \in var\_mut$ of the $j-th$ population vector, where $j \in pop\_mut$, a random $\varepsilon$ value drawn from a uniform distribution in the close interval $[-1,1]$, that is $P_{ji}=P_{ji}+\varepsilon .$ We include to the population ${\mathcal {P}}$ only the feasible new vectors, which updated dimension $dim\_new\_pop2$ could be grater than $dim\_new\_pop$.
  - **Step 4.2** We then mutate the last $GPS+SP+SPR(P+1)+RP$ components associated with the security levels in the network, of all the *feavec* vectors. If $P_{ij}<1, \quad \forall i=1, ... dim\_new\_pop2,$ $\forall j=1,\ldots ,GSP+SPR(P+1)$, then we replace the corresponding $\sigma$ variable (as seen in the Crossover Step), with random $\varepsilon _{mut} \in [0,10/D_g].$ We include in ${\mathcal {P}}$ the mutate feasible vectors.
- **Step 5. Stop Criterion.** The cycle 2-4 steps are repeated $niter=25$ times or until the difference between the actual best objective function and the previous cycle best objective function is less than $tolerance=10^{-5}$. Afterwards, the best solution in ${\mathcal {P}}$ is returned as the result.

Algorithm 1 shows a pseudocode of this heuristic.
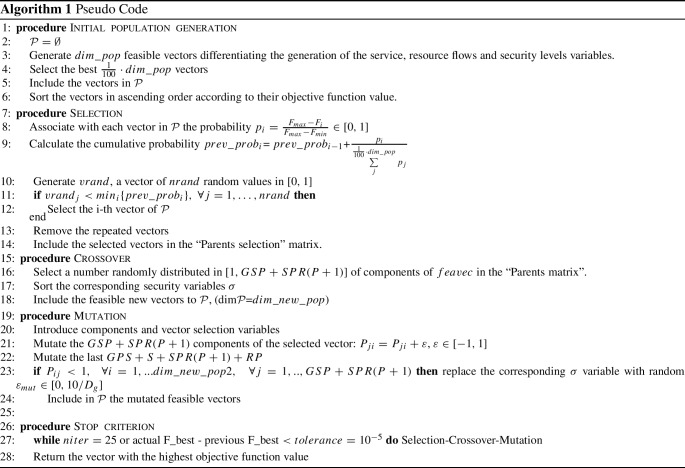


### Illustrative computational experiments

In this section, we assess the heuristic algorithms. In order to perform the algorithm and to illustrate the Mathematical Model results, the 5G Network configuration selected for the computational experiments is simple, as next showed in Subsection 5.1.1. The illustrative configuration instance is solved by the new heuristic method proposed, as well as the standard genetic algorithm method Davis (1991) and by the exact method (Interior-Point Algorithm, see Byrd et al. (2000) and Waltz et al. (2006)). We compare the performance in terms of the best objective function relative percent difference (RPD) value of our heuristic with the exact method . We also compare the performance in terms of execution time only of our heuristic with the GA method. The algorithms were coded using Matlab and were run on an HP laptop with an AMD compute cores 2C+3G processor, 8 GB RAM.

#### Configuration

To test our heuristic algorithm as previously mentioned, we considered a very simple configuration, which can be referred to a small coverage area, in order to clearly illustrate the mathematical model and the results. Other authors have also referred to simple numerical examples with a small number of UAVs, although in different contexts or with different objective functions (see, for example, Dayarian et al. (2020),Gao et al. (2021), Ramirez-Atencia et al. (2017), Wu et al. (2018) ). Therefore, the size and the data of the computational experiments are chosen for easy interpretation purposes and we consider the following 5G network configuration. The network consists of $G=1$ service, executed in $S=1$ slice. This service can be provided by $P=2$ providers. To execute the service $R=2$, resources are needed. Moreover, solving large instances of the 5G network, with exact method, implies expensive cost in terms of CPU time. For this reason, the 5G network configuration is chosen and here illustrated has a simple topology. The numerical data are constructed for easy interpretation purposes and read as follows:

$$\begin{aligned} D_{11}=5,\ D_{12}=7, D_1=30, \gamma _{11}=1,\ \gamma _{21}=0.5, \\ B_1=150,\ B_2=200,\ \alpha ^{(1)}=80, \alpha ^{(2)}=50, \\ A_{11}=30,\ A_{21}=32,\ A_{12}=27,\ A_{22}=26, \\{\overline{C}}_{11}=60 ,\ {\overline{C}}_{12}=75,\ \rho _{min}=12, \\ \beta _{11}=1.5,\ \beta _{21}=1.7,\ \beta _{1111}=1.9,\ \beta _{1121}=2.1, \\ \beta _{1112}=1.9,\ \beta _{1122}=2.1,\ \beta '_{11}=1.2,\ \beta _{111}=1.8, \\ \beta _{12}=1.5,\ \beta _{22}=1.7,\ \beta _{1211}=1.9,\ \beta _{1221}=2.1, \\ \beta _{1212}=1.9,\ \beta _{1222}=2.1,\ \beta '_{12}=1.2,\ \beta _{112}=1.8. \end{aligned}$$

**Table 2:** Coefficients for resources transmission/transport cost functions

$r=1$			$r=2$		
$\mu _{1{\tilde{p}}1p}$	$p=1$	$p=2$	$\mu _{1{\tilde{p}}2p}$	$p=1$	$p=2$
${\tilde{p}}=1$	0.1	0.1	${\tilde{p}}=1$	0.9	0.9
${\tilde{p}}=2$	0.2	0.2	${\tilde{p}}=2$	0.7	0.7
$\mu '_{1{\tilde{p}}1p}$	$p=1$	$p=2$	$\mu '_{1{\tilde{p}}2p}$	$p=1$	$p=2$
${\tilde{p}}=1$	0.5	0.5	${\tilde{p}}=1$	0.45	0.45
${\tilde{p}}=2$	0.1	0.1	${\tilde{p}}=2$	0.35	0.35

The cost functions are chosen polynomial as follows:

$$\begin{aligned}&c_{11{\tilde{p}}}(x_{11{\tilde{p}}})=\eta _{11{\tilde{p}}}\cdot (x_{11{\tilde{p}}})^2+\eta '_{11{\tilde{p}}}\cdot (x_{11{\tilde{p}}}),\\&\forall {\tilde{p}}=1,2, \end{aligned}$$

where $$\eta _{111}=0.2$$, $$\eta _{111}'=0.1$$, $$\eta _{112}=0.1$$ and $$\eta _{112}'=0.25$$,

$$\begin{aligned}&c_{1{\tilde{p}}rp}(y_{1p{\tilde{p}}1p})=\mu _{1{\tilde{p}}rp}\cdot (y_{1p{\tilde{p}}rp})^2+\mu '_{1{\tilde{p}}rp}\cdot (y_{1p{\tilde{p}}rp}),\\&\forall r=1,2,\ \forall {\tilde{p}}=1,2,\ \forall p=1,2, \end{aligned}$$

where the respective coefficients are reported in Table 2,

$$\begin{aligned}&c_{1{\tilde{p}}rp}^{(A)}(y_{1{\tilde{p}}rp})=\delta _{1{\tilde{p}}rp}\cdot (y_{1{\tilde{p}}rp})^2+\delta '_{1{\tilde{p}}rp}\cdot (y_{1{\tilde{p}}rp}),\\ {}&\forall r=1,2,\ \forall {\tilde{p}}=1,2,\ \forall p=1,2, \end{aligned}$$

where $\delta _{1112}=0.1$, $\delta _{1211}=0.2$, $\delta _{1122}=0.1$, $\delta _{1221}=0.2$ and $\delta '_{1{\tilde{p}}rp}=0$, for all ${\tilde{p}},\ r,\ p$,

$$\begin{aligned} c_{r{\tilde{p}}}^{(E)}=\lambda _{r{\tilde{p}}}\cdot \left( \sum _{p=1}^2y_{1pr{\tilde{p}}}\right) ^2+\lambda '_{r{\tilde{p}}}\cdot \left( \sum _{p=1}^2y_{1pr{\tilde{p}}}\right) ,\quad \forall {\tilde{p}}=1,2, \end{aligned}$$

where $\lambda _{11}=0.2$, $\lambda _{21}=0.3$, $\lambda _{12}=0.4$, $\lambda _{22}=0.5$, $\lambda '_{r{\tilde{p}}}=0$, for all $r,\ {\tilde{p}}$ and, finally,

$$\begin{aligned} c_{1{\tilde{p}}}^{(E)}=\kappa _{1{\tilde{p}}}\cdot (x_{11{\tilde{p}}})^2+\kappa '_{1{\tilde{p}}}\cdot (x_{11{\tilde{p}}}),\quad \forall {\tilde{p}}=1,2, \end{aligned}$$

where $$\kappa _{11}=0.1$$, $$\kappa _{12}=0.2$$ and $$\kappa '_{11}=\kappa '_{12}=0$$.

**Fig. 4:**
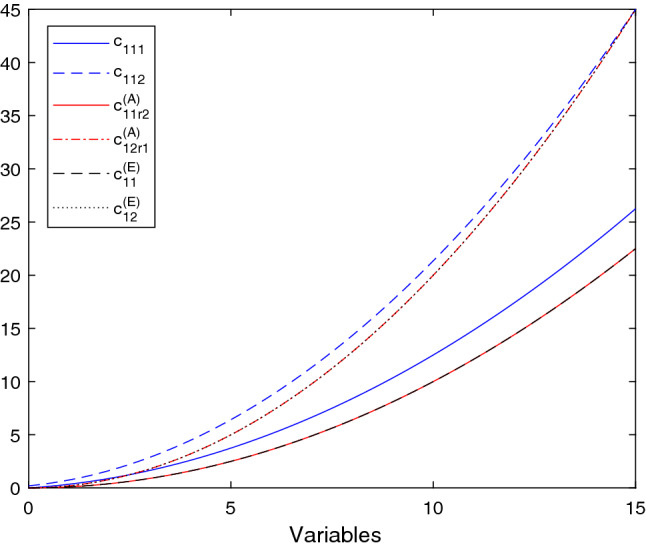
Transport/transmission costs, rental costs, and utilization/execution cost

**Fig. 5:**
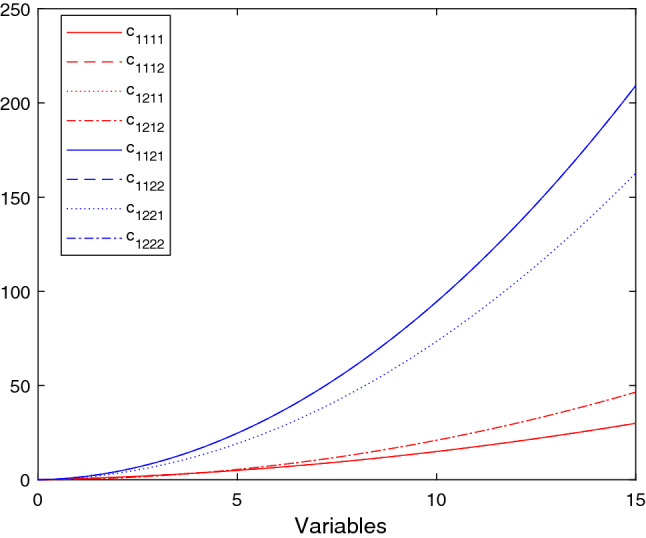
Transport/transmission costs of resources from providers to slices

**Fig. 6:**
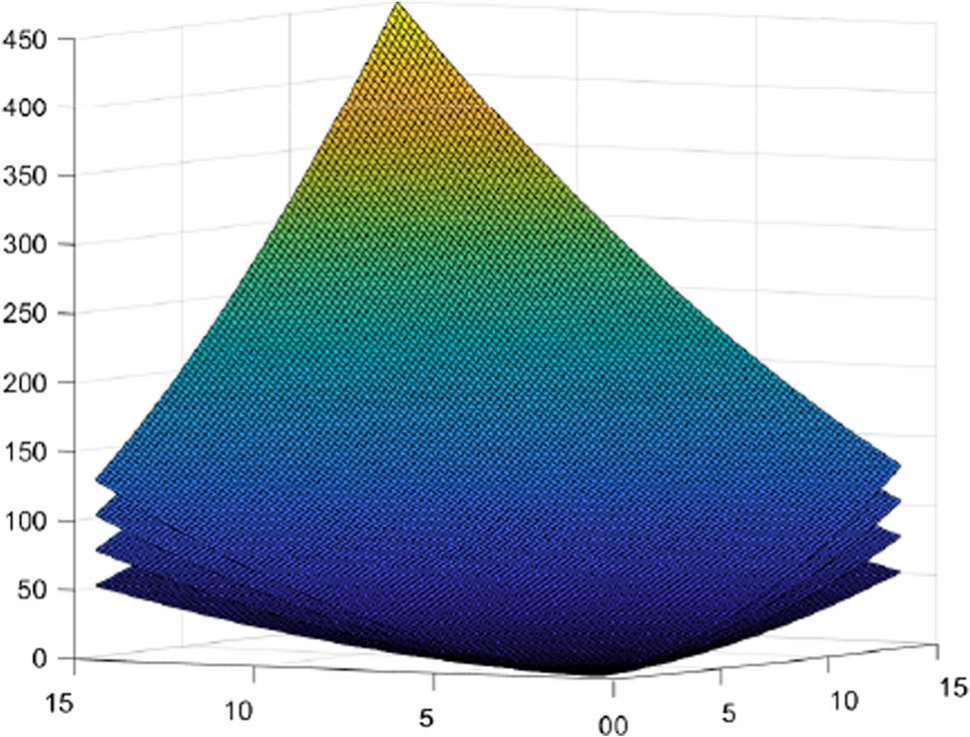
Utilization/execution costs incurred by each provider to use/execute all his resources ($c_{r{\tilde{p}}}^{(E)}$)

See Fig. 4, Figs. 5 and Fig. 6 for the trend of the transport/transmission costs, rental costs, and utilization/execution costs, the transport/transmission costs of resources from providers to slices and the utilization/execution costs incurred by each provider to use/execute all his resources. Note that we have differentiated the figures based on the size and dimensions of the chart.

#### Calibration and evaluation

In order to choose the optimal value for the parameter *dim_pop* of the heuristic algorithm, we perform an analysis of experiments to find such optimal value. The parameter is tested over the following values:

$$\begin{aligned} dim\_pop\in&\{500,\;1000,\;2000,\;3000,\;4000,\;5000,\;6000,\\&\;7000,\;8000,\;9000,\;10000,\;11000,\;12000\}, \end{aligned}$$

having in total 13 different values. The lowest value tested for the $dim\_pop$ parameter was chosen considering that, based on the initial experience, a good solution quality was not obtained for any lower values. The largest value tested for the $dim\_pop$ was chosen so that the computational times would not be too large. The response variable considered is the Relative Percent Deviation (RPD), defined for each instance (each $dim\_pop$ parameter) as follows:

$$\begin{aligned} {RPD}=\frac{F_{opt}-F_{New}}{F_{opt}}\times 100 \%, \end{aligned}$$

where $F_{opt}$ is the value of the objective function calculated for the optimal solutions obtained with the exact method, and $F_{New}$ is the value of the objective function calculated for the solutions found by the new heuristic proposed.

**Fig. 7:**
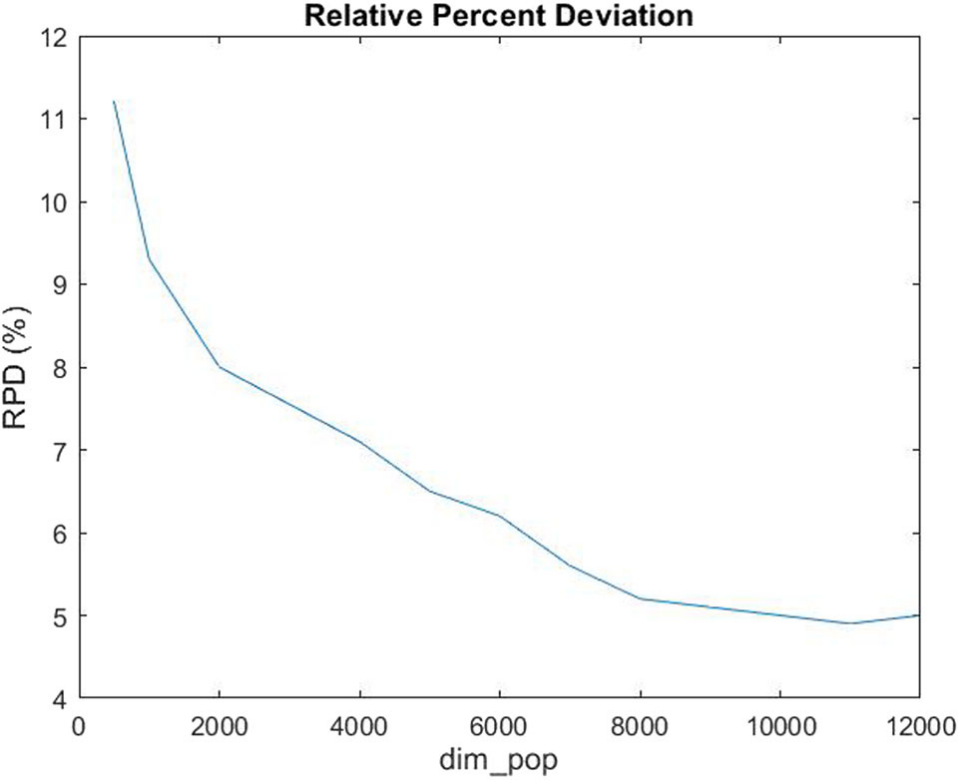
Mean Relative Percent Deviation varying $dim\_pop$

Figure 7 shows how the mean *RPD* is decreasing as the number of the population $dim\_pop$ increases. Specifically, we note that from 10000 onwards, the mean Relative Percent Deviation settles at about $5\%$. The same percentage gap was found in Murray and Raj (2020) by Murray et al., where the solutions provided by the proposed genetic algorithm were compared with the optimal solutions provided by a MILP problem solved via Gurobi. This comparison was made for a network with 1-, 2-, 3- and 4 UAVs. Particularly, in each of the configurations a percentage error of 5.0 %, 4.9 %, 4.7 % and 5.4 %, respectively, was obtained. Instead, Zhang et al. proposed a new algorithm whose relative percent deviation, between the proposed algorithm and the optimal one, is $24.98\%$ (see Figure 5 in Zhang et al. (2018)). Moreover, authors in Zhang et al. (2018) also compared their algorithm with the greedy scheme and with a local search heuristic called Kariz proposed in Ghaznavi et al. (2017) where the *RPD* equals $69.60\%$. Therefore, the novel heuristic algorithm proposed in this paper, compared with those in the literature, appears to be efficient and accurate.

**Fig. 8:**
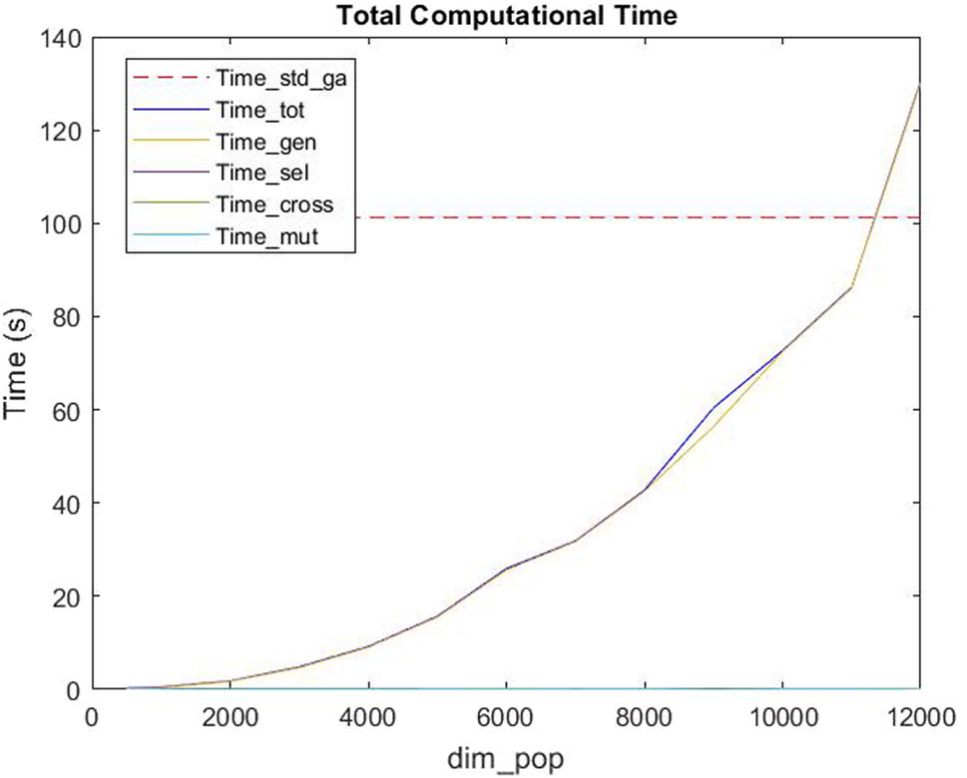
Computational times varying $dim\_pop$

**Fig. 9:**
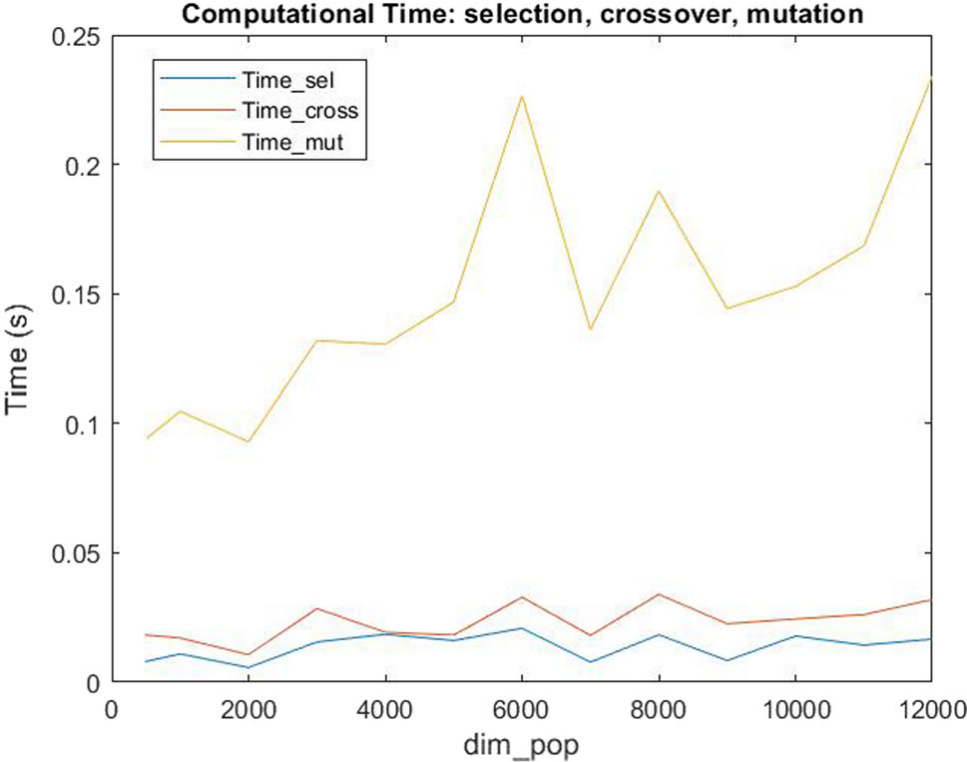
Computational Time of Selection, Crossover and Mutation

We underline that the main component of the total computational time is given by the needed time for the generation of the population. Indeed, as shown in Fig. 9 the computational times for selection, crossover and mutation are always less than 0.25 seconds. Therefore, we have analysed the computational times varying the $dim\_pop$ parameter and we noted that the total computational time $Time\_tot$ (as $Time\_gen$, the time for the generation) increases as the number of the dimension $dim\_pop$ increases. We also observe that the mean computational time required to solve the problem with the standard genetic algorithm is $Time\_std\_ga=101.18s$, and that, if the population’s dimension does not exceed 11000, the total computational time to solve the problem with our new heuristic proposed is less than $Time\_std\_ga$ (see Fig. 8). Therefore, we conclude that 11000 is a good population’s dimension both for *RPD* and computational time evaluations. Moreover, the Hybrid MOGA-CSP algorithm proposed by Ramirez et al. spent from 3min 5s to 26min 43s runtime for each execution in which the number of generations needed to converge for each dataset varies from 12 to 122 (see Table 11 in Ramirez-Atencia et al. (2017)), while we noted that the new algorithm proposed in this paper needs only from 2 to 8 (with an average value of 5) generations to converge and spent less total runtime. Furthermore, we analysed *niter* and *tolerance*, because, as previously described, the algorithm repeats the Selection, Crossover and Mutation procedures *niter* times or until the tolerance (given by the difference between the last two best objective functions obtained) is less than a very small value, that we established as $1\times 10^{-5}$. We considered it appropriate to set the *niter* value at 25, since it is big enough to meet the required tolerance (indeed, from computational experiments we noted that the number of iterations is usually less than 5) and, at the same time, does not exceed $Time\_std\_ga$.

**Fig. 10:**
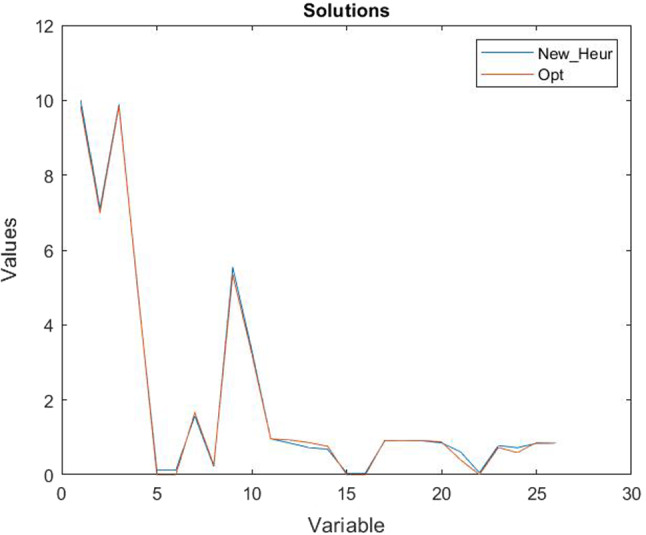
Comparison between the solutions obtained with the new heuristic and the optimal ones

The comparison between the solutions obtained with our new heuristic algorithm (with the parameters previously analysed) and the exact algorithm is depicted in Fig. 10, and we can observe that the solutions are almost all the same, only some variables differ, but very lightly. We also underline that all the constraints (4)–(11) are always satisfied. Other observations that we can draw from Fig. 10 are about the variables and their optimum values obtained in the numerical example. The second variable, namely $x_{112}$, has a value slightly greater than its minimum value, $D_{12}$. This can be explained by observing that provider 2 has higher costs than provider 1; therefore, the service requests handled by provider 2 will only be those of its exclusive customers, as established by the constraint (5), while all the remaining requests are handled by the most convenient provider, that is provider 1. The third and ninth variables are, according to constraint (8), slightly lower than the first and second variables, respectively, since the quantity of resource $r = 1$ needed to execute a unit of service is $\gamma _ {11} = 1$. Analogously, the fourth and tenth variables are slightly lower than half of the first and second variables, respectively, since the quantity of resource $r = 2$ needed to execute a unit of service is $\gamma _ {21} = 0.5$. The fifth to eighth variables are close to 0 because the cost of using the resources of the other providers is higher than the cost of using their own resources. Therefore, the resources of other providers are used (with the lowest cost) only if necessary. Finally, we observe that the remaining variables (from the eleventh onwards) take values from 0 to 1 since they are related to security levels which, as previously mentioned (see, for example constraint (11), they cannot have a value greater than or equal to 1.

**Fig. 11:**
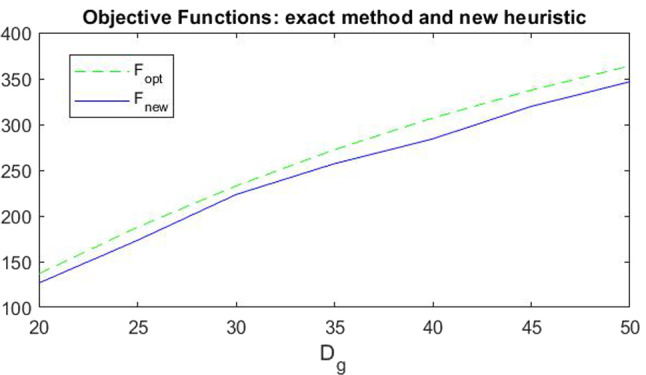
Comparison between the objective functions obtained with the new heuristic and the optimal ones

**Fig. 12:**
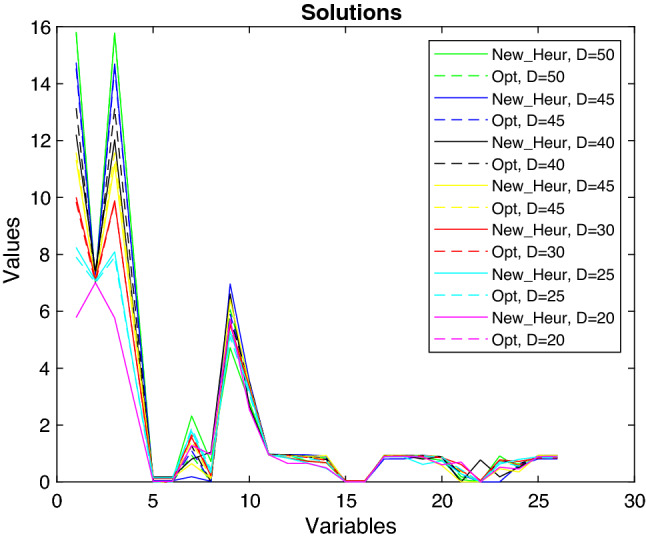
Comparison between the solutions obtained with the new heuristic and the optimal ones varying the flow of requests

For completeness, we tested the new proposed heuristic for different values of the service request $D_g=\{20, 25,30, 35, 40, 45, 50\}$ (these values are chosen so that the problem admits feasible solutions). Figure 11 shows the values of the objective functions obtained by using the new heuristic and the exact method for each of the service request values. It is easy to note that despite the variability range of the variables increases as the request $D_g$ increases, the distance between the two curves does not vary much and is constant enough as well as the computational time that is always less than $Time\_std\_ga$. Moreover, Fig. 12 shows the trend of the variables obtained both with the exact method (the optimal variables) and with our new heuristic. We can observe that, although the values are different, the trend remains unchanged (as explained for Fig. 10), even if the demand, that is the requests for services, increases.

## Conclusion

In this paper, a constrained optimization problem describing the provision of services in a 5G network architecture consisting of a multi-level network has been developed. Service providers try to maximize their profits, given by the difference between the revenues obtained from the sale of services and the rent of their own resources and the costs associated with the rental of resources and with the transmission/transport of resources and services, determining the optimal flows of resources and services between the network levels and their own optimal security levels in order to minimize the expected financial damage associated with a successful cyberattack. Furthermore, the analysed context is a supply chain network, where some UAVs are used to execute services, allowing the providers to extend the 5G network, thanks to the virtualization, one of the main characteristics of the 5G technology. For the resolution of the numerical experiments, a new genetic algorithm was proposed. Its main phases, such as the initial population generation, selection, crossover and mutation, were inspired by the nature of the theoretical mathematical model. This new algorithm has been compared with the standard genetic algorithm on various configurations, and a greater efficiency in terms of computational times was found. Furthermore, the results obtained through the new algorithm were compared with the exact Interior-Point Algorithm, obtaining a good estimate of the optimal exact results. The model previously described can certainly be extended. In our future work, we are going to study a more comprehensive model, in which we introduce a bigger area to be covered (intended as a union of small areas) and a set of Time Slots and in which we investigate the impacts of the size of the area and a more general case of multi-hop communication between UAVs in the same network. Therefore, we intend to test the proposed new heuristic solving numerical examples on large and real instances (of which we are collecting data).

## Data Availability

Enquiries about data availability should be directed to the authors.
